# Comparative effectiveness of aerobic, resistance, and combined training on cardiovascular disease risk factors: A randomized controlled trial

**DOI:** 10.1371/journal.pone.0210292

**Published:** 2019-01-07

**Authors:** Elizabeth C. Schroeder, Warren D. Franke, Rick L. Sharp, Duck-chul Lee

**Affiliations:** 1 Department of Kinesiology and Nutrition, University of Illinois, Chicago, IL, United States of America; 2 Department of Kinesiology, Iowa State University, Ames, IA, United States of America; Weill Cornell Medical College Qatar, QATAR

## Abstract

Although exercise has well-documented health benefits on cardiovascular disease (CVD), the benefit of combination exercise on CVD risk factors in individuals with elevated risk has not been fully elucidated. We compared the effects of aerobic, resistance, and a combination of both aerobic and resistance training on CVD risk factors including peripheral and central BP, cardiorespiratory fitness (CRF), muscular strength, body composition, blood glucose and lipids. Sixty-nine adults (58±7 years) with an elevated blood pressure or hypertension, overweight/obesity, and sedentary lifestyle were randomized to one of the three 8-week exercise programs or a non-exercise control group. Participants in all three exercise groups had an equal total exercise time, 3 days/week (aerobic: 60 minutes/session vs. resistance: 60 minutes/session vs. combination: aerobic 30 minutes/session plus resistance 30 minutes/session). Combined training provided significant reductions in peripheral (-4 mmHg) and central diastolic BP (-4 mmHg), increase in CRF (4.9 ml/kg/min), increase in upper (4 kg) and lower (11 kg) body strength, and increase in lean body mass (0.8 kg) (p <0.05). Aerobic training only increased CRF (7.7 ml/kg/min), and reduced body weight (-1.0 kg) and fat mass (-0.9 kg) (p <0.05). Resistance training only increased lower body strength (13 kg) and reduced waist circumference (-1.7 cm) (p <0.05). However, neither aerobic or resistance training alone showed significant reductions in BP (p>0.05). Furthermore, a composite score of CVD risk factors indicated a greater reduction with combination training compared to the control group. In conclusion, among individuals at an increased risk for CVD, as little as 8-weeks of combined training may provide more comprehensive CVD benefits compared to time-matched aerobic or resistance training alone.

## Introduction

Hypertension, or elevated blood pressure, leads to increased risk of cardiovascular disease (CVD) and is the number one leading risk factor for mortality [1]. However, hypertension is also one of the most significant modifiable risk factors in the prevention of cardiovascular disease [2]. With a prevalence of hypertension in the US population around 29% [3] and its predicted continual increase [4], it is a critical health concern to reduce hypertension and improve cardiovascular disease risk. Although pharmacological interventions are often relied on to reduce blood pressure, lifestyle modification is the first line of therapy suggested by several governing bodies [5–7].

Lifestyle modifications often emphasize improvements in diet and exercise habits. The American College of Sports Medicine (ACSM), the American Heart Association, and others [8] have all provided professional exercise recommendations for adults with hypertension. However, most of these recommendations focus on aerobic exercise prescription [8]. The health benefits of aerobic exercise are well established [9–11], but less data exist in regards to the health benefits of resistance exercise, especially on cardiovascular health in individuals with elevated blood pressure [12]. Indeed, recent reviews and meta-analyses have established that aerobic and resistance exercise can both have a significant blood pressure lowering effect of approximately 3–4 mmHg in both systolic and diastolic blood pressure [13–16]. This small decrease has shown to be highly clinically relevant as it is estimated to reduce cardiac morbidity by 5%, stroke by 8–14%, and all-cause mortality by 4% in the average population.[13] Most earlier exercise studies on blood pressure and other CVD risk factors, however, have focused more on either aerobic or resistance training alone.

Recent studies have begun using a combination of aerobic and resistance exercise to determine if additive benefits exist in which an ~3 mmHg reduction in blood pressure has been observed [17]. Although a similar reduction in blood pressure compared to either aerobic or resistance training alone, both aerobic and resistance exercise provide independent, modality specific benefits toward cardiovascular disease risk factors. In general, aerobic exercise induces greater improvements in cardiorespiratory fitness and cardio-metabolic variables, whereas resistance exercise mainly effects muscular strength and has positive effects on body composition, such as muscle mass and bone density.

The combination of aerobic and resistance exercise could have an additive effect and further decrease the risk of CVD risk factors, however, there is a vast breadth of populations and co-morbidities included in studies on combination training, various exercise prescriptions and timing, and few with the sole focus on blood pressure reduction [17]. Additionally, many of the combined exercise training studies do not have an aerobic- or resistance-only group or are not a well-controlled randomized trials [15]. Therefore, it is unclear whether the additional benefits of combination exercise is simply due to extra exercise time rather than the independent additive benefits from each aerobic and resistance exercise. Further, data on well-controlled exercise interventions in middle-aged individuals with a heightened cardiovascular disease risk profile without overt disease are still scarce.

The purpose of this study was to compare the effects of time-matched aerobic training only, resistance training only, and combined aerobic and resistance training on blood pressure and CVD risk factors compared to a non-exercising control group. We conducted an 8-week randomized controlled trial in middle-aged adults with an elevated blood pressure or hypertension, overweight or obesity, and sedentary lifestyle. We hypothesized the combined aerobic and resistance training would elicit greater improvement in blood pressure and CVD risk factors compared with either training only group.

## Methods

### Participants

This study consisted of 69 adults, 45 to 74 years of age, who had an elevated blood pressure or hypertension (systolic/diastolic blood pressure of 120-149/80-99 without taking anti-hypertensive medications), overweight or obesity (body mass index [BMI] of 25–40 kg/m^2^), and sedentary lifestyle (not meeting the aerobic and resistance exercise guidelines over the last 3 months [18]). Participants were free of any serious medical conditions (unstable coronary heart disease, decompensated heart failure, severe pulmonary hypertension) that would not allow safe participation in exercise according to ACSM and American Heart Association [19,20]. Other exclusion criteria included individuals who smoked; pregnant women or women anticipating pregnancy; and those who planned on being away for more than 2 weeks during the intervention period. The Iowa State University Institutional Review Board (IRB) approved this study (IRB ID: 14–330; August 8^th^, 2014) and each participant signed an informed consent document prior to participation. The protocol was registered with ClinicalTrials.gov (ID: NCT03734146; https://clinicaltrials.gov/ct2/show/NCT03734146?id=NCT03734146&rank=1) following completion of the study, as it was considered an internally funded pilot study. The authors confirm that all ongoing and related trials for this intervention are registered.

### Study design

Study recruitment was completed between August and October 2014. To enhance adherence, participants were screened by phone prior to enrollment (by ECS) in an orientation session. After determining that study criteria were met, participants attended two education sessions to minimize dropout. Participants were then randomly assigned by a study statistician to one of four parallel groups in a 1:1:1:1 ratio: 1) no-training control, 2) aerobic training only, 3) resistance training only, or 4) combination of both aerobic and resistance training. Group allocation was based on age, sex, BMI, and baseline blood pressure and completed using a computer-generated randomization. Participants were not aware of their group allocation until baseline measures were completed. This was a single-center, parallel-group, superiority study conducted at Iowa State University in Ames, Iowa, United States from August to December 2014.

Outcome measures were assessed at baseline (August to October 2014) and following the 8-week intervention (October to December 2014). Participants arrived to the laboratory for baseline and 8-week follow-up measures having refrained from physical activity for at least 24 hours. All pre- and post-intervention measures were conducted in the same laboratory at the same time of day in an identical sequence at each time point by investigators blinded to group allocation. Measures occurred over two days. On the first visit, participants arrived to the lab following an overnight fast (>12 hours) for assessment of blood pressure, heart rate, body composition, lipid profile, and glucose. Participants were then familiarized with the treadmill and strength tests. On the second visit, participants completed assessments of cardiorespiratory fitness and muscular strength. A 3-day diet record was kept for the next 3-days. Participants additionally wore a pedometer throughout the entire study duration to monitor outside physical activity levels.

Full details of the trial protocol can be found in the supporting information (S1 Protocol) available with the full text of this article at http://journals.plos.org/plosone/.

### Assessments

Peripheral and central blood pressure and resting heart rate were measured using the Sphygmocor XCEL (AtCor Medical, Itasca, IL, USA) automated oscillometric device. A brachial blood pressure cuff was placed on the participant’s left arm over the brachial artery in a seated position. The brachial pressure was measured 3 times by the device, with a two-minute rest period between each measurement. Immediately following each blood pressure, the brachial volume displacement waveform was obtained by inflating the cuff to a sub-diastolic pressure. A generalized transfer function was used to estimate central blood pressure [21]. The first reading was discarded and the device reported the average of the last 2 readings for all measurements.

BMI (kg/m^2^) was calculated using measured body weight and height. Waist circumference was measured at the level of the umbilicus (cm). Body composition was assessed via multi-frequency bioelectrical impedance analysis (BIA) with 8 tactile electrodes (InBody 720, Biospace Co, Ltd, Seoul, Korea), in which variables of body fat percentage, fat mass, and fat free mass were obtained.

Cardiorespiratory fitness was assessed using a submaximal treadmill exercise test following the modified Balke and Ware protocol [22]. All participants reached 70% of their heart rate reserve (equivalent to 85% of age-predicted maximal heart rate) prior to ending the submaximal test. Cardiorespiratory fitness was estimated using the following formula from the American College of Sports Medicine: 3.5 + (0.1 x speed) + (1.8 x speed x grade) [19].

Maximal contractile strength was assessed with a seated chest and leg press (TechnoGym Wellness System, Gambettola, Italy) 1 repetition maximum (RM) following standard procedures [19]. Participants warmed up with light resistance and weight was added at 5–10 kg (or 5–10% of body weight) increments for upper body and 15–20 kg (or 10–20% of body weight) increments for lower body until a maximum load was reached. A 2 minute resting period was allowed between each attempt. An absolute 1 RM was determined when the participant successfully lifted the weight through the entire range of motion but could no longer increase the load. For one participant who exceeded the maximum amount of weight on the leg press, the 1 RM was estimated using a training load chart [23].

A 5-mL venous blood draw from a superficial arm vein was obtained to assess lipid profile (total cholesterol, low- and high-density lipoprotein cholesterol, and triglycerides) and glucose. Samples were collected in a serum separation tube and centrifuged for 15 minutes following a 30-minute clotting time. Serum samples were analyzed offsite by LabCorp (Des Moines, IA).

### Exercise intervention

All exercise groups completed 8 weeks of supervised training with equal training time, exercising 3 days per week for 60 minutes per session. The non-exercise control group did not exercise during the intervention.

The aerobic only group utilized the treadmill or cycle ergometer. Starting at 40% of their heart rate reserve, participants were progressed to approximately 70% of their heart rate reserve (equivalent to 85% of the age-predicted heart rate maximum). Participants could choose to exercise at a higher intensity but not to exceed 80% of their heart rate reserve. A heart rate monitor was worn during all exercise sessions.

The resistance only group performed 12 exercises: chest press, shoulder press, pull-down, lower-back extension, abdominal crunch, torso rotation, biceps curl, triceps extension, leg press, quadriceps extension, leg curl, and hip abduction. The program started with 2 sets of 18–20 maximal repetitions and progressed to 3 sets of 10–14 maximal repetitions with a rest of 1–2 minutes between sets. In this program, participants achieved exhaustion in each set, indicating the lower the repetition, the higher the intensity. Total weight lifted in each exercise session was automatically monitored and stored by a computer-controlled exercise intervention system (TechnoGym Wellness System).

The combination group completed 30 minutes of aerobic exercise and 30 minutes of resistance exercise per session. Participants followed the same intensity and protocol as the aforementioned individual groups, but the resistance training was reduced to 8 exercises instead of 12 (excluding shoulder press, arm curl, arm extension, and leg extension) and 2 sets instead of 3. Order was not specified, although a majority of participants completed aerobic exercise prior to resistance. All participants were asked to refrain from any moderate or vigorous physical activity outside the intervention, and reported daily steps using an accelerometer (OMRON HJ-321, OMRON Healthcare, Hoofddorp, Netherlands) during the entire intervention period.

### Dietary counseling

All study groups received the same dietary counseling by a registered dietician based on the Dietary Approaches to Stop Hypertension (DASH) Diet [24] to minimize dietary variability among groups [25]. The focus of this counseling was on changing the quality of the diet without changing the total energy intake to avoid weight loss. A 3-day food diary was obtained during the first and eighth week of the intervention and analyzed using The Food Processor Diet and Nutrition Analysis Software (ESHA, Salem, Oregon).

### Data analysis

A composite risk factor score was derived by summing the standardized residuals (Z-scores) of the change value from baseline to follow-up for 5 well-established CVD risk factors, also recently identified by the American Heart Association [26]: mean arterial pressure, total cholesterol, lower body strength, cardiorespiratory fitness, and body fat percentage [10,27,28]. Lower body strength and cardiorespiratory fitness were reverse coded (multiplied by -1) prior to entry in the equation, due to increases in strength and fitness seen as beneficial. A lower score is indicative of a better CVD risk factor profile following the intervention. We also explored other composite risk factor scores based on different combinations of CVD risk factors. This was a pilot study funded internally by the institution (Iowa State University), thus power and sample size were not officially calculated, but determined by the amount of the awarded research fund, scope of the project, and recommendations by the expert project application review committee.

All data were checked for normality and transformed when necessary. Descriptive statistics were calculated for each variable and presented as mean (standard deviation, SD). The primary outcome variables were peripheral and central systolic and diastolic blood pressure. Secondary outcomes included BMI, weight, waist circumference, body composition, cardiorespiratory fitness, muscular strength, and fasting lipids and glucose. Analyses were performed on an intention-to-treat basis using the last observation carried forward method and included all randomly allocated persons at baseline. A linear-mixed effects model was used to assess the change in all outcome variables with repeated measures for time, group, and time-by-group interaction, adjusted for age, sex, and the baseline value of each outcome variable. A covariance structure was determined the best fit with the lowest values for the information criteria after evaluation of different structures. A Bonferroni correction was applied to correct for multiple comparisons between groups. Data are presented as least-squares adjusted means with standard error (SE) or mean change with 95% confidence intervals (CI). Statistical analyses were performed using the SAS software (SAS Institute, Cary, NC). All p-values are 2-sided, with significance set a priori at p <0.05.

## Results

**Fig 1:**
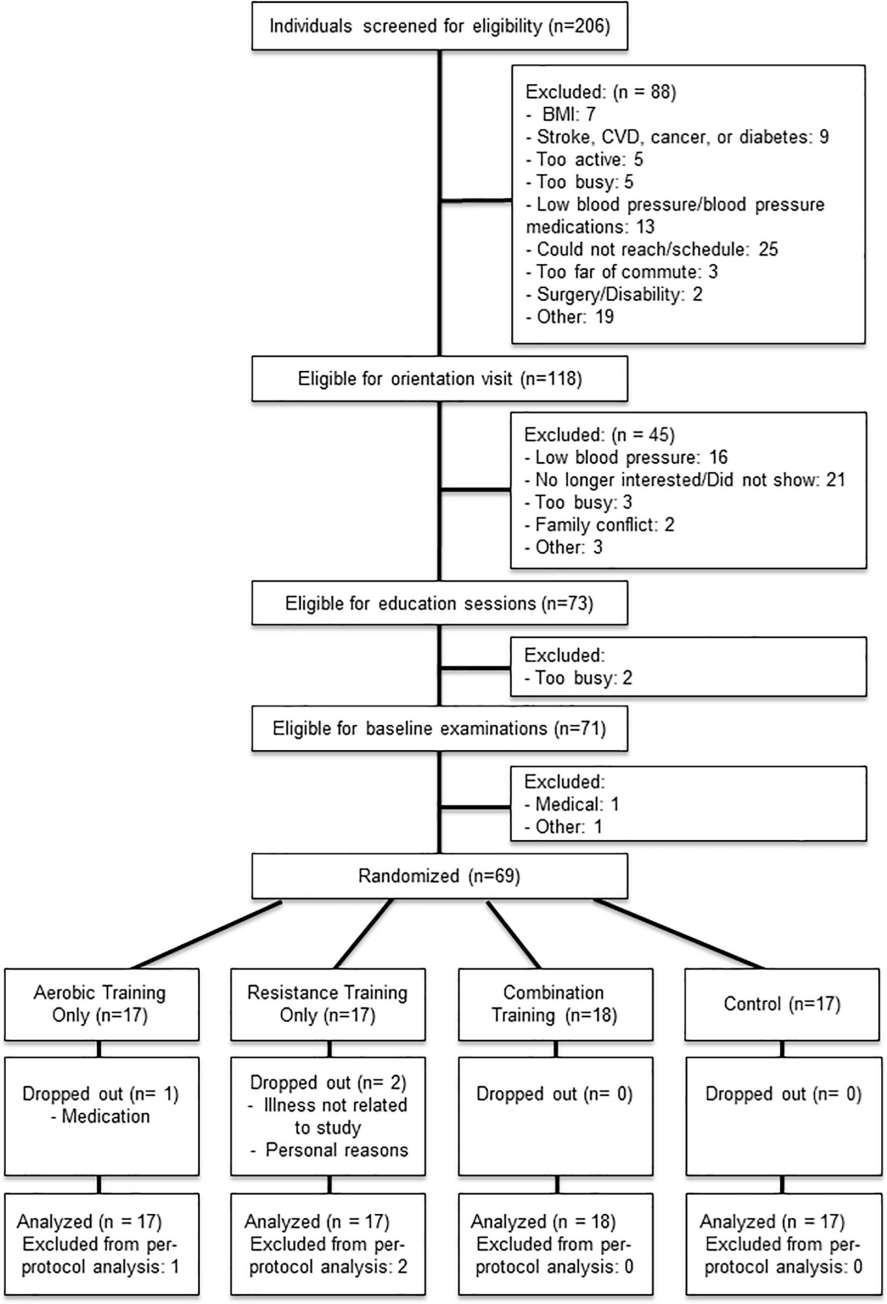
Participant flow chart.

Fig 1 shows the flow of participants from recruitment to follow-up. Of the 69 individuals randomized, 66 (96%) completed the 8-week intervention. Mean exercise attendance was 96% in all groups except resistance training (92%). On average, aerobic exercise participants completed 100 ± 6% of the prescribed exercise amount in minutes, with a mean exercise intensity greater than what was prescribed (119 ± 13%) based on the heart rate measured during each exercise session throughout the intervention. Resistance training participants completed 100 ± 2% of prescribed sets, and exercised at the weight prescribed 99 ± 11% of the time. Based on pedometer counts, lifestyle physical activity outside the intervention did not change significantly over time (5689 ± 2005, 5429 ± 1883, 5404 ± 2125, 5664 ± 2285, 5466 ± 1896, 5535 ± 2116, 5191 ± 1893, and 5095 ± 2039 steps/day from week 1 to week 8, respectively; p = 0.69) and no difference was observed across groups (p>0.05). Also, no significant changes were noted in total calorie, fat, carbohydrate, protein, or sodium intake (p>0.05). No adverse events occurred in any of the intervention groups.

At baseline, participants were 58 ± 7 years old, 61% women, and had a body mass index of 32.4 ± 5.2 kg/m^2^. Baseline resting systolic blood pressure was 131 ± 13 and diastolic blood pressure was 81 ±9 mmHg (Table 1).

**Table 1:** Baseline participant characteristics^a^.

	All	Aerobic	Resistance	Combination	Control
N	69	17	17	18	17
Age, years	58 (7)	58 (7)	57 (9)	58 (7)	58 (6)
Women, n (%)	42 (61%)	10 (59%)	10 (59%)	11 (61%)	11 (65%)
Post-Menopausal, n (%)	35 (83%)	9 (90%)	7 (70%)	9 (82%)	10 (91%)
Race/ethnicity					
White, n (%)	64 (93%)	16 (94%)	16 (94%)	16 (89%)	16 (94%)
Non-White, n (%)	5 (7%)	1 (6%)	1 (6%)	2 (11%)	1 (6%)
Body Composition					
BMI, kg/m^2^	32.4 (5.2)	32.5 (5.9)	33.1 (5.9)	31.9 (5.5)	32.4 (3.7)
Weight, kg	94.5 (19.0)	97.1 (20.7)	95.8 (21.2)	93.6 (18.9)	91.4 (16.0)
Waist Circumference, cm	105 (13)	103 (14)	106 (17)	104 (13)	106 (10)
Lean Body Mass, kg	56.9 (13.2)	59.2 (13.4)	58.2 (13.9)	55.9 (14.0)	54.2 (12.0)
Fat Mass, kg	38.3 (11.4)	38.6 (13.0)	38.3 (12.6)	38.5 (12.5)	37.8 (7.3)
Body Fat Percentage, %	40.1 (8.1)	39.1 (8.6)	39.5 (8.0)	40.6 (10.0)	41.4 (5.7)
Resting Hemodynamics					
Resting Heart Rate, bpm	69 (9)	67 (10)	70 (10)	66 (7)	72 (10)
Peripheral SBP, mmHg	131 (13)	131 (10)	131 (14)	131 (16)	129 (12)
Peripheral DBP, mmHg	81 (9)	81 (10)	81 (11)	81 (10)	80 (8)
Central SBP, mmHg	120 (11)	120 (10)	122 (11)	121 (13)	119 (12)
Central DBP, mmHg	82 (9)	82 (10)	82 (11)	82 (9)	82 (7)
VO_2_max, ml/kg/min	30.6 (9.3)	31.1 (9.5)	29.9 (8.9)	31.4 (11.4)	29.9 (7.6)
Lower Body 1 RM, kg	123 (47)*	122 (44)	136 (56)	111 (43)	123 (44)*
Upper Body 1 RM, kg	44 (21)	46 (23)	45 (21)	42 (20)	45 (23)
Fasting Blood Lipids and Glucose					
Glucose, mg/dL	98 (8)*	97 (8)	102 (8)*	98 (8)	96 (6)
Triglycerides, mg/dL	162 (73)	151 (68)	167 (93)	146 (67)	184 (60)
HDL Cholesterol, mg/dL	53 (13)*	55 (13)	49 (12)	52 (15)*	54 (13)
LDL Cholesterol, mg/dL	130 (32)*	134 (28)	121 (33)*	130 (37)	133 (28)
Total Cholesterol, mg/dL	214 (37)	219 (26)	200 (33)	215 (47)	223 (36)
Diet	(n = 64)	(n = 15)	(n = 15)	(n = 18)	(n = 16)
Total Intake, kcal	1882 (473)	1966 (582)	1857 (484)	1842 (465)	1871 (387)
Fat, g	73 (21)	75 (20)	68 (20)	69 (22)	78 (22)
Protein, g	79 (22)	77 (21)	80 (24)	80 (26)	79 (16)
Carbohydrates, g	229 (76)	250 (101)	230 (66)	228 (67)	210 (66)
Sodium, mg	2952 (1024)	2928 (1036)	2821 (1073)	2959 (1132)	3091 (916)

BMI: body mass index; DBP: diastolic blood pressure; HDL: high-density lipoprotein cholesterol; LDL: low-density lipoprotein cholesterol; RM: repetition maximum; SBP: systolic blood pressure

^a^:Data presented as mean (SD) for continuous variables or number of participants (%) for categorical variables

*: Missing one data point

Table 2 presents the data on the primary outcome variables. Following 8 weeks of exercise, the combination group reduced peripheral and central diastolic blood pressure -4 mmHg [95% CI: -6, -1] and -4 mmHg [95% CI: -7, -2], respectively. No significant improvements were observed in peripheral or central systolic blood pressure in any group. However, the aerobic and combination groups both showed a reduction in resting heart rate of -2 bpm [95% CI: -5, 1], which were significantly different from the control group (p<0.02).

**Table 2:** Baseline, follow-up, and changes in resting hemodynamics*.

		Mean (SE)		Mean (95% CI)		
Intervention Group	n	Baseline Value	Follow-up Value	Within-Group Changes	Between-Group Comparison vs. Control Group	P-Value vs. Control Group
Peripheral Systolic Blood Pressure						
Aerobic	17	131 (3)	131 (3)	0 (-4, 4)	1 (-5, 7)	0.72
Resistance	17	131 (3)	130 (3)	-1 (-5, 3)	0 (-6, 5)	0.95
Combination	18	131 (3)	131 (3)	0 (-4, 4)	1 (-5, 6)	0.74
Control	17	129 (3)	129 (3)	-1 (-5, 3)	-	
Peripheral Diastolic Blood Pressure						
Aerobic	17	81 (2)	79 (2)	-2 (-4, 0)	-2 (-6, 1)	0.20
Resistance	17	80 (2)	80 (2)	0 (-2, 3)	0 (-4, 3)	0.91
Combination	18	81 (2)	77 (2)	**-4 (-6, -1)**	**-4 (-7, 0)**	**0.04**
Control	17	80 (2)	80 (2)	0 (-2, 3)	-	
Central Systolic Blood Pressure						
Aerobic	17	120 (3)	119 (3)	-1 (-4, 3)	0 (-4, 5)	0.88
Resistance	17	122 (3)	119 (3)	-2 (-6, 1)	-1 (-6, 4)	0.62
Combination	18	121 (3)	120 (3)	-1 (-4, 3)	0 (-4, 5)	0.88
Control	17	119 (3)	118 (3)	-1 (-4, 3)	-	
Central Diastolic Blood Pressure						
Aerobic	17	82 (2)	79 (2)	-2 (-5, 0)	-2 (-5, 2)	0.39
Resistance	17	81 (2)	81 (2)	0 (-3, 2)	1 (-3, 4)	0.66
Combination	18	82 (2)	78 (2)	**-4 (-7, -2)**	**-3 (-7, 0)**	**0.05**
Control	17	82 (2)	81 (2)	-1 (-3, 2)	-	
Resting Heart Rate						
Aerobic	17	67 (2)	65 (2)	-2 (-5, 1)	**-5 (-9, -1)**	**0.01**
Resistance	17	69 (2)	72 (2)	2 (-1, 5)	0 (-4, 4)	0.82
Combination	18	66 (2)	64 (2)	-2 (-5, 1)	**-5 (-9, 1)**	**0.02**
Control	17	73 (2)	74 (2)	2 (-1, 5)	-	

*All values adjusted for age, sex, and baseline value of each hemodynamic outcome measure.

Secondary outcomes of body composition, fitness, strength, and lipid profile are summarized in Table 3. Aerobic training yielded the greatest benefit in body composition with reductions in BMI (-0.3 kg/m^2^ [95% CI: -0.7, 0.0]), weight (-1.0 kg [95% CI: -1.9, -0.1]) and fat mass (-0.9 kg [95% CI: -1.5, -0.2]). The resistance training group decreased waist circumference by -1.7 cm [95% CI: -3.3, -0.1] while the combination group increased both lean body mass (0.8 kg [95% CI: 0.1, 1.5]) and weight (0.9 kg [95% CI: 0.00, 1.8]). Improvements in both cardiorespiratory fitness and muscular strength were seen in the combined training group, with expected training benefits in aerobic and resistance training only. Cardiorespiratory fitness was increased in the aerobic training group and combination group by 7.7 ml/kg/min [95% CI: 3.9, 11.5] and 4.9 ml/kg/min [95% CI: 1.1, 8.7], respectively. However, no significant changes were observed in either the resistance or control group. Lower body muscular strength increased significantly in both the resistance (13 kg [95% CI: 4, 23]) and combination training groups (11 kg [95% CI: 2, 20]) in comparison with the control group. Upper body muscular strength increased in all exercise groups. Minimal changes occurred in fasting glucose and the lipid profile following training. The resistance training group reduced triglycerides (-26 mg/dL [95% CI: -47, -5]), as did the control group (-22 mg/dL [95% CI: -43, -1]).

**Table 3:** Changes in body composition, cardiorespiratory fitness, muscular strength, blood glucose and lipids*.

Characteristics	Aerobic	Resistance	Combination	Control
Body Composition				
BMI, kg/m^2^	**-0.3 (-0.7, 0.0)**^e^	-0.1 (-0.5, 0.2)	0.2 (-0.1, 0.6)	0.0 (-0.3, 0.4)
Weight, kg	**-1.0 (-1.9, -0.1)**^e^	-0.2 (-1.1, 0.7)	**0.9 (0.0, 1.8)**	0.1 (-0.8, 1.0)
Waist Circumference, cm	0.4 (-1.2, 2.0)	**-1.7 (-3.3, -0.1)**^c^^e^	0.9 (-0.7, 2.5)	0.5 (-1.2, 2.1)
Lean Body Mass, kg	-0.3 (-1.0, 0.5)	0.1 (-0.6, 0.9)	**0.8 (0.1, 1.5)**^b^^c^	-0.2 (-0.9, 0.6)
Fat Mass, kg	**-0.9 (-1.5, -0.2)**^b^	-0.3 (-1.0, 0.3)	-0.1 (-0.7, 0.5)	0.2 (-0.5, 0.8)
Body Fat, %	-0.5 (-1.1, 0.0)	-0.2 (-0.8, 0.4)	-0.5 (-1.0, 0.1)^b^	0.2 (-0.4, 0.8)
Cardiorespiratory Fitness and Muscular Strength				
VO_2_max, ml/kg/min	**7.7 (3.9, 11.5)**^b^^d^	1.5 (-2.4, 5.4)	**4.9 (1.1, 8.7)**	1.9 (-1.8, 5.8)
Lower Body 1 RM, kg	-1 (-10, 8)	**13 (4, 23)**^c^	**11 (2, 20)**^c^	2 (-7, 12)
Upper Body 1 RM, kg	**4 (2, 6)**	**4 (2, 6)**	**4 (3, 6)**	**2 (0, 4)**
Blood Glucose and Lipids				
Glucose, mg/dL	0 (3, 3)	-1 (-4, 2)	-2 (-4, 1)	2 (-1, 5)
Triglycerides, mg/dL	-11 (-32, 10)	**-26 (-47, -5)**	3 (-17, 24)	**-22 (-43, -1)**
HDL Cholesterol, mg/dL	0 (-2, 2)	0 (-2, 3)	-2 (-4, 0)	-2 (-4, 1)
LDL Cholesterol, mg/dL	-1 (-9, 6)	-1 (-9, 7)	2 (-6, 9)	3 (-4, 11)
Total Cholesterol, mg/dL	-4 (-12, 5)	-6 (-15, 2)	-3 (-11, 5)	-3 (-11, 6)

BMI, body mass index; HDL, high-density lipoprotein; LDL, low-density lipoprotein; RM, repetition maximum*All data presented as mean (95% confidence interval) and adjusted for age, sex, and baseline value of each outcome measure

^a^Different from all other groups, p<0.05

^b^Different from control group, p<0.05

^c^Different from aerobic training group, p<0.05

^d^Different from resistance training group, p<0.05

^e^Different from combination training group, p<0.05

Following the exercise intervention, the composite cardiovascular disease risk score indicated a slight reduction, although not significant, in the aerobic (-0.34 [95% CI: -1.46, 0.77]), resistance (-0.10 [95% CI: -1.25, 1.06]), and combination (-0.78 [95% CI: -1.89, 0.34]) training groups and increase in the control group (0.95 [95% CI: -0.20, 2.10]). In comparison, the reduction for the combination group was different from the control group (p = 0.04). We found similar results in other composite risk factor scores based on different CVD risk factors.

## Discussion

A specific goal of this study was to ensure that the total exercise time was consistent across exercise groups to be more applicable to the general population and help determine the effectiveness of each intervention style. The primary finding of this study was that only combined training provided significant changes in blood pressure, with reductions in diastolic pressure. Although participants in the aerobic and resistance training groups did receive benefit from exercise training in other aspects of cardiovascular health (i.e. body composition), the combined group experienced more cumulative benefits across all cardiovascular outcomes as indicated by the composite score. Despite aerobic exercise having the most well-known health benefits, this study supports the 2008 Physical Activity Guidelines recommendation of combination training and suggests individuals may receive greater and more complete CVD health benefits by performing both aerobic and resistance exercise.

Our training intervention was effective as we observed training-induced adaptations commonly reported after aerobic and resistance training: aerobic training led to significant increases in cardiorespiratory fitness and resistance training led to significant increases in muscular strength. Both aerobic and combination training had significant increases in cardiorespiratory fitness in this study, and the aerobic group had a larger increase in cardiorespiratory fitness than the combination group, although not statistically significant (effect size = 0.34). However, it is important to note that only the combined training group experienced significant benefits in both cardiorespiratory fitness and muscular strength in this study. While each exercise intervention elicited the expected changes in fitness and strength, the primary outcome of this study focused on blood pressure and CVD risk factors.

Our exercise intervention did not result in systolic blood pressure reductions. Meta-analyses and earlier studies have reported decreases in systolic blood pressure following aerobic or resistance exercise training alone or in combination [17,29–32]. This contradiction may reflect that most exercise interventions that elicit reductions in blood pressure have been at least 12 weeks long [30,33–35]. This could indicate that an 8-week intervention is not long enough to detect the expected blood pressure responses to exercise in this specific population. Additionally, participants qualified for the study based on orientation screening blood pressures, however, during the baseline visit 15 participants had a blood pressure <120/80. With normal baseline blood pressure, reductions with exercise are less likely to be detected [17] and could be another potential explanation to our results, although these participants were equally spread throughout each intervention group. Despite no changes in systolic blood pressure, significant reductions in diastolic blood pressure were seen with combination training.

Despite minimal changes in blood pressure, these data contribute to a growing body of literature. A recent meta-analysis was performed on randomized controlled trials looking at resistance training only in pre- and hypertensive individuals [12]. Only 5 studies were available, in which 4 had a population over the age of 60 years. Although many trials have been performed with aerobic training in this population, this stresses the importance of performing well-controlled resistance and combination exercise interventions in a middle-aged population with elevated risk factors without overt disease.

Exercise in conjunction with changes in body composition have previously shown benefits for blood pressure reduction and other CVD risk factors [36]. In regards to body composition, the aerobic training group had the most significant improvements with reductions in BMI, weight, fat mass, and body fat percentage. A recent meta-analysis of randomized controlled trials comparing aerobic, resistance, and combination training found aerobic training resulted in greater fat mass reductions than resistance training [37]. Although not significant, all training groups had a reduction in body fat percentage, which lends support to exercise training as an effective intervention for healthy weight management. Combination training increased weight slightly but elicited beneficial changes in body composition (increase in lean body mass). Overall, training-induced changes in fasting blood lipids and glucose were small and did not vary between training and control groups. It was not surprising that fasted lipids and glucose were unaltered by exercise training considering all groups were within normal ranges at baseline.

The primary outcome of our paper was blood pressure reduction, however, the study of a single CVD risk factor does not fully elucidate the cumulative benefit of exercise on multiple aspects of CVD risk studied, such as blood pressure, body composition, fitness, strength, and metabolism. Furthermore, individuals who do not show improvement in one CVD risk factor may show improvements in other CVD risk factors. One way to assess overall risk is to create a composite score using the sum of standardized scores [38,39]. Our composite score suggested that combination training elicits greater overall improvements in CVD risk factors than the control group. This provides support for the prescription of combination training for the greatest overall benefits from exercise training in middle-aged adults with elevated CVD risk.

Strengths of this study include the randomization that ensured participants were comparable at baseline in major confounding factors (e.g., age, sex), orientation sessions to minimize potential drop-out that led to high attendance and adherence, intention-to-treat analysis to include all participants randomized at baseline, and participants at high risk for developing CVD. Additionally, unlike previous studies [25,40], all exercise sessions had equal training time with objective verification of the amount and intensity of exercise being completed. Objective measurement of all exercises in this study eliminated the possibility of over- or under-reporting. Furthermore, the exercise prescriptions were well tolerated by sedentary, overweight/obese individuals, making them easily obtainable by a more general population. Lastly, participants wore a pedometer throughout the intervention period to minimize the uncertainty about changes in physical activity outside the intervention, commonly seen in other studies.

A randomized clinical trial is not without its limitations. One limitation comes from cardiorespiratory fitness being estimated using a submaximal treadmill test instead of a maximal effort. Although these values may be overestimated at baseline and with the change following training, the consistency in measurement technique still allows for a comparison to be made between groups and with training. Our control group was also not a true non-treatment group since they received the lifestyle educational session and information regarding diet prior to starting. Furthermore, although the reported steps and diet did not change significantly throughout the study, volunteers for an exercise intervention tend to be a motivated group. Thus, subtle lifestyle changes that could not be accounted for in this study may have contributed to our findings.

## Conclusion

Among individuals at high risk of developing CVD with an elevated blood pressure or hypertension, a combination of aerobic and resistance exercise training resulted in improved diastolic blood pressure, increased lean body mass, and increased strength and cardiorespiratory fitness, even though the exercise intervention was only 8 weeks long. Moreover, these data suggest that combination training may be of better value than either aerobic or resistance training alone, as it appeared to have the most beneficial effect on the composite of CVD risk factors. However, further studies with a larger sample size and longer intervention is clearly warranted on the overall cardiovascular benefits of different types and combinations of exercise. Cumulatively our data suggest that concurrent aerobic and resistance training may be a more potent means to improve CVD risk factor burden among at-risk middle-aged adults than aerobic or resistance exercise alone.

## Supporting information

S1 CONSORT Checklist (DOC)

S1 Protocol Institutional Review Board approved protocol. (PDF)
