# Supplementary material for: Comparative effectiveness of aerobic, resistance, and combined training on cardiovascular disease risk factors: A randomized controlled trial
Source: PLoS One. 2019 Jan 7;14(1):e0210292. doi: 10.1371/journal.pone.0210292 (PMC6322789; doi:10.1371/journal.pone.0210292)
Supplement: S1 Protocol — (PDF) [file pone.0210292.s002.pdf]

IOWA STATE UNIVERSITY
OF SCIENCE AND TECHNOLOGY

Institutional Review Board
Office for Responsible Research
Vice President for Research
1138 Pearson Hall
Ames, Iowa 50011-2207
515 294-4566
FAX 515 294-4267

Date: 7/17/2014

To: Dr. Duck-Chul Lee
251 Forker Bldg

CC: Lorraine Lanningham-Foster
220 MacKay Hall

From: Office for Responsible Research

Title: Independent and Combined Effects of Aerobic and Resistance Training on Blood Pressure

IRB ID: 14-330

Approval Date: 7/15/2014 **Date for Continuing Review:** 7/14/2016

Submission Type: New **Review Type:** Full Committee

The project referenced above has received approval from the Institutional Review Board (IRB) at Iowa State University according to the dates shown above. Please refer to the IRB ID number shown above in all correspondence regarding this study.

To ensure compliance with federal regulations (45 CFR 46 & 21 CFR 56), please be sure to:

- **Use only the approved study materials** in your research, **including the recruitment materials and informed consent documents that have the IRB approval stamp.**
- **Retain signed informed consent documents for 3 years after the close of the study**, when documented consent is required.
- **Obtain IRB approval prior to implementing any changes** to the study by submitting a Modification Form for Non-Exempt Research or Amendment for Personnel Changes form, as necessary.
- **Immediately inform the IRB of** (1) **all serious and/or unexpected adverse experiences** involving risks to subjects or others; and (2) **any other unanticipated problems involving risks** to subjects or others.
- **Stop all research activity if IRB approval lapses**, unless continuation is necessary to prevent harm to research participants. Research activity can resume once IRB approval is reestablished.
- **Complete a new continuing review form** at least three to four weeks prior to the **date for continuing review** as noted above to provide sufficient time for the IRB to review and approve continuation of the study. We will send a courtesy reminder as this date approaches.

Please be aware that IRB approval means that you have met the requirements of federal regulations and ISU policies governing human subjects research. **Approval from other entities may also be needed.** For example, access to data from private records (e.g. student, medical, or employment records, etc.) that are protected by FERPA, HIPAA, or other confidentiality policies requires permission from the holders of those records. Similarly, for research conducted in institutions other than ISU (e.g., schools, other colleges or universities, medical facilities, companies, etc.), investigators must obtain permission from the institution(s) as required by their policies. **IRB approval in no way implies or guarantees that permission from these other entities will be granted.**

Upon completion of the project, please submit a Project Closure Form to the Office for Responsible Research, 1138 Pearson Hall, to officially close the project.

Please don't hesitate to contact us if you have questions or concerns at 515-294-4566 or IRB@iastate.edu.

IRB ID: 14-330

INSTITUTIONAL REVIEW BOARD (IRB)
Application for Approval of Research Involving Humans

RECEIVED
JUN ~~16~~ 17 (RS) 2014
By IRB

Title of Project: Independent and Combined Effects of Aerobic and Resistance Training on Blood Pressure		

Principal Investigator (PI): Duck-chul Lee		Degrees: Ph.D.
University ID: 937139203	Phone: 515-294-8042	Email Address: dclee@iastate.edu
Correspondence Address: 251 Forker Building, Ames, IA 50011		
Department: Kinesiology		College/Center/Institute: College of Human Sciences

PI Level: ☒ Tenured, Tenure-Eligible, & NTER Faculty ☐ Adjunct/Affiliate Faculty ☐ Collaborator Faculty ☐ Emeritus Faculty ☐ Visiting Faculty/Scientist ☐ Senior Lecturer/Clinician ☐ Lecturer/Clinician, w/Ph.D. or DVM ☐ P&S Employee, P37 & above ☐ Extension to Families/Youth Specialist ☐ Field Specialist III ☐ Postdoctoral Associate ☐ Graduate/Undergrad Student ☐ Other (specify:)

FOR STUDENT PROJECTS *(Required when the principal investigator is a student)*		
Name of Major Professor/Supervising Faculty:		
University ID:	Phone:	Email Address: @iastate.edu
Campus Address:		Department:
Type of Project (check all that apply):	☐ Thesis/Dissertation	☐ Class Project ☐ Other (specify:)

Alternate Contact Person: Lorraine Lanningham-Foster	Email Address: lmlf@iastate.edu
Correspondence Address: 220 MacKay Hall	Phone: 515-294-4684

ASSURANCE

- I certify that the information provided in this application is complete and accurate and consistent with any proposal(s) submitted to external funding agencies. Misrepresentation of the research described in this or any other IRB application may constitute non-compliance with federal regulations and/or academic misconduct.
- I agree to provide proper surveillance of this project to ensure that the rights and welfare of the human subjects are protected. I will report any problems to the IRB. See Reporting Adverse Events and Unanticipated Problems for details.
- I agree that modifications to the approved project will not take place without prior review and approval by the IRB.
- I agree that the research will not take place without the receipt of permission from any cooperating institutions when applicable.
- I agree to obtain approval from other appropriate committees as needed for this project, such as the IACUC (if the research includes animals), the IBC (if the research involves biohazards), the Radiation Safety Committee (if the research involves x-rays or other radiation producing devices or procedures), etc., and to obtain background checks for staff when necessary.
- I understand that IRB approval of this project does not grant access to any facilities, materials, or data on which this research may depend. Such access must be granted by the unit with the relevant custodial authority.
- I agree that all activities will be performed in accordance with all applicable federal, state, local, and Iowa State University policies.

이덕철 6/17/2014

Signature of Principal Investigator Date

Signature of Major Professor/Supervising Faculty Date
(Required when the principal investigator is a student)

- I have reviewed this application and determined that departmental requirements are met, the investigator(s) has/have adequate resources to conduct the research, and the research design is scientifically sound and has scientific merit.

PHILIP E. MARTIN

Printed Name of Department Chair/Head/Director

Philip E Martin 6/17/14

Signature of Department Chair/Head/Director Date

**For IRB Use Only**		Full Committee Review: ☑	Review Date: July 15, 2014
		EXPEDITED per 45 CFR 46.110(b):	Approval/Determination Date: July 15, 2014
Approval Not Required:	☐	Category Letter	Approval Expiration Date: July 14, 2016
Not Research:	☐	EXEMPT per 45 CFR 46.101(b):	
No Human Subjects:	☐	Not Approved: ☐	Risk: Minimal ☑ More than Minimal ☐
IRB Reviewer's Signature	Kerry A Agnitsch	July 17, 2014	

Research Involving Humans Study Information

Please provide answers to all questions, except as specified. Incomplete forms will be returned without review.

PART A: KEY PERSONNEL

1. **List all members and relevant qualifications of the project personnel and define their roles in the research.** Key personnel include the principal investigator, co-principal investigators, supervising faculty member, and any other individuals who will have contact with the participants or the participants' data (e.g., interviewers, transcribers, coders, etc.). This information is intended to inform the committee of the training and background related to the specific procedures that each person will perform on the project. For more information, please see Human Subjects – Persons Required to Obtain IRB Training.

NAME	Interpersonal contact or communication with subjects, or access to private identifiable data?	Involved in the consent process?	Contact with human blood, specimens, or other biohazardous materials?	Other Roles in Research	Qualifications (i.e., special training, degrees, certifications, coursework, etc.)	Human Subjects Training Date
Duck-chul Lee	☒	☒	☒	PI	PhD	3/16/2011
Lorraine Lanningham-Foster	☒	☒	☒	co-PI	PhD	12/11/2008
Nathan Meier	☒	☒	☒	GRA	PhD Candidate	9/10/2011
Elizabeth Schroeder	☒	☒	☒	GRA	MS Candidate	8/24/2013
Maren Wolff	☒	☒	☒	GRA	PhD Candidate	6/3/2009
	☐	☐	☐			
	☐	☐	☐			
	☐	☐	☐			
	☐	☐	☐			
	☐	☐	☐			
	☐	☐	☐			
	☐	☐	☐			
	☐	☐	☐			
	☐	☐	☐			
	☐	☐	☐			
	☐	☐	☐			
	☐	☐	☐			

Please complete additional pages of key personnel as necessary.

PgP 3-30 received 7/9/14 (SP)

☐ Yes ☒ No	**2.**	**Does your study include children (persons under age 18) as research subjects?**
		If *Yes*, please read and respond to the following:<br><br>ISU policy requires that background checks be completed for all researchers and key personnel who will have any contact with children involved in this research project. Details regarding this policy can be found here. **Principal Investigators and faculty supervisors are responsible** for ensuring that background checks are completed BEFORE researchers or key personnel may have any contact with children. Records documenting completion of the background checks must be kept with other research records (e.g., signed informed consent documents, approved IRB applications, etc.) and may be requested during any audits or Post-Approval Monitoring of your study.
☐ **Agreed**	**2.a.**	**Please check here to indicate that you have read this information and agree that you will comply with these requirements.**

PART B: FUNDING INFORMATION AND CONFLICTS OF INTEREST

☐ Yes ☒ No	**1.**	**Is or will the project be externally funded?**
		If *No*, skip to question 8.
		If *Yes*, please identify the type(s) of source(s) from which the project is directly funded.<br><br>☐ Federal agency<br>☐ State/local government agency<br>☐ University or school<br>☐ Foundation<br>☐ Other non-profit institution<br>☐ For-profit business<br>☐ Other; specify:
☐ Yes ☐ No	**2.**	**Is ISU considered to be the Lead or Prime awardee for this project?**
☐ Yes ☐ No	**3.**	**Are there or will there be any subcontracts issued to others for this project?**
☐ Yes ☐ No	**4.**	**Is or will this project be funded by a subcontract issued by another entity?**
☐ Yes ☐ No	**5.**	**If ISU is the recipient of the subcontract, does it involve any federal funding, such as federal flow-through funds?**
**6.**		**If this project will be externally funded, please provide the complete name(s) of the funding source(s); please do not use acronyms. If any subcontracts will be issued to others, please describe and include a list of all entities.**
☐ **Attached**	**7.**	**Please attach a <u>complete and final copy</u> of the entire grant proposal or contract from which the project is or will be funded.**

[illegible]

☐ Yes ☒ No	8.	**Do or will any of the investigators or key personnel listed on this application have a conflict of interest management plan in place with the Office of the Vice President for Research & Economic Development?**

PART C: GENERAL OVERVIEW – PURPOSE AND EXPECTED BENEFITS

1. Research Objectives – Briefly explain in language *understandable to a layperson* the purpose and specific aim(s) of the study.

We will compare the effects of aerobic exercise, resistance exercise, and a combination of both on blood pressure.

Primary Aim 1: We will determine the independent and combined associations of aerobic and resistance exercise on resting blood pressure.
Hypothesis 1: After 12 weeks of exercise intervention, the combination of aerobic and resistance exercise training group will have a greater reduction in blood pressure than the aerobic training only and resistance training only groups.
Hypothesis 2: After 12 weeks of exercise intervention, the aerobic training only and resistance training only groups will have comparable reductions in blood pressure compared with the no training control group.

2. Broader Impacts/Significance – Explain in language *understandable to a layperson* why this research is important and how the information gained in this study is expected to advance knowledge and/or serve the good of society.

Although blood pressure reduction by exercise appears modest, as little as 2-3 mmHg blood pressure reduction may decrease coronary heart disease by 5%, stroke by 10%, and all-cause mortality by 4% in the general population. Regarding cardiovascular disease (CVD) prevention, few studies have evaluated the effect of resistance exercise, independent of and combined with aerobic exercise. This study may contribute to transforming the paradigms of current physical activity research and clinical exercise programs, which focus primarily on aerobic exercise for health. Therefore, this study potentially has a profound impact on developing effective CVD prevention strategies.

☒ Yes ☐ No	3.	**Benefits to Participants –** Are there any expected direct benefits to research participants from participation in the research? **Note:** Monetary compensation is ***not*** considered to be a benefit of participation in research.

If *Yes,* please describe the expected benefits to participants.

With the 12 weeks of exercise training, participants may improve their cardiorespiratory fitness and muscular strength. Other benefitis may be seen by a reduction in risk for hypertension, diabetes, hypercholesterolemia, and other chronic diseases known to be reduced by physical activity and exercise.

PART D: PARTICIPANT SELECTION

1. How many individuals do you plan to include in the study (including those involved in screening procedures)? The number listed here is the maximum number of participants that may be included in the study.

In this randomized controlled trial, we plan to recruit 120 participants before screening sessions to allow for the fact that some of the participants may not qualify after screening. In this way, we expect to include at least 80 participants for randomization after screening procedures. A majority of the participants will be recruited from a current database of >400 adults who voluntarily participated in our previous survey study and have agreed to be contacted for future studies, as well as an e-mail sent to Iowa State University staff and faculty. Flyers (attached) will also be posted around Iowa State University as a means of recruitment.

2. Inclusion Criteria – Describe the specific characteristics of persons that will be included in your study, and provide justification for these requirements.

*Men and women aged 45-74 years because they are expected to get the most cardiovascular health benefits from exercise.

*Participants with systolic/diastolic blood pressure of 120-159/80-99 mm Hg who are not on anti-hypertension medication because it is necessary to include participants whose blood pressure is both 1) high enough to provide a likilhood that exercise may have a detectable benefit, 2) low enough to ensure anti-hypertensive medication is not required thoughout the study, and 3) broad enough to prevent major barriers to recruitment. Power computations suggest that in order to have adequate power to detect improvements in systolic blood pressure, a reduction of 4, and preferably 5, mmHg is necessary. This magnitude of improvement would be unlikely unless participants with elevated blood pressure were accepted into the study. Also, recruitment would be compromised severely with an upper limit of 139 mmHg. The overall referral rate for hypertension is likely to be low in this 12-week exercise intervention study, because of the extensive baseline evaluations and lifestyle intervention, and the probable hypotensive effect of regular exercise. Several large exercise intervention studies (Blumenthal, JAMA, 1991; Church, JAMA, 2007; and Stewart, Arch Intern Med, 2005) included participants with blood pressure of 120-159/80-99, and reported no major adverse events. Although it is challenging to include a physician as a key personnel in this study due to limited budget and the large study sample, we will refer any participants who are at high risk of potential adverse events and need use of antihypertensive medication to their physician based on their medical history and fitness levels before and throughout the study. In addition, participants with blood pressure 140-159/90-99 mmHg will be refered to their physician for approval to participate in the study before randomization and exercise intervention. Furthermore, to reduce any potential risk, we will follow our strict intervention programs such as 3 education sessions, personalized exercise program, and supervision by certified exercise trainers throughout the study. Participants will also be allowed to withdraw at any time.

*Non-smokers because smoking has strong effects on blood pressure and other cardiovascular disease risk factors, possibly becoming a confounder on the outcome variable of interest (blood pressure).

*Overweight or obese individuals (body mass index of 25-40 kg/m2) because they are at an elevated risk for cardiovascular disease, thus expected to get the most health benefits from this study. In addition, more than two thirds of Americans are overweight or obese.

*Sedentary individuals (not meeting the aerobic or resistance exercise guidelines of <500 MET-minutes/week of aerobic exercise, which is equivalent to 150 minutes per week of moderate-intensity aerobic activity and <2 days per week of resistance exercise over the last 3 months) because we would like to be able to attribute any change to the new exercise regimen.

3. Exclusion Criteria – Describe the characteristics of persons who will not be allowed to participate in your study, and provide justification for their exclusion.

Exclusion criteria will include any serious medical problems that prevent participants from exercising according to the American College of Sport Medicine and American Heart Association guidelines about contraindications to exercise including:

* Unstable coronary heart disease or decompensated heart failure
* Severe pulmonary hypertension or aortic stenosis
* Acute myocarditis, endocarditis, pericarditis, or aortic dissection
* Other medical condition that is life-threatening or that can interfere or be aggravated by the exercis training because these serious diseases can affect the participant's exercise behavior and blood pressure.

Plans to be away >2 weeks during the 3 month intervention period because missing the training program for longer than 2 weeks may affect the the relationship between the exercise protocol and blood pressure.

Premenopausal women or postmenopausal women with hormonal replacement therapy because those hormones may have an effect on blood pressure and other cardiovascular disease risk factors. Pregnant women or anticipated pregnancy via IVF or other medical procedures during the course of the intervention because the changes in hormone levels and physiological status may affect their blood pressure.

4. Do you intend, or is it likely, that your study will include any persons from the following vulnerable populations? (Check all that apply.)

- ☐ Children (any persons under age 18, including ISU/college students who may be under age 18)
  Specify age range:
- ☐ Prisoners
- ☐ Persons with impaired decision-making capacity, such as those with dementia or severe cognitive impairment, those declared incompetent, persons in life-threatening situations, etc.
- ☐ Wards of the State
- ☐ Persons who are institutionalized
- ☐ Pregnant women or fetuses
- ☐ Neonates
- ☐ Educationally disadvantaged
- ☐ Economically disadvantaged
- ☐ Students in a class taught by the researchers
- ☐ Employees or subordinates of the researchers
- ☐ Other vulnerable population, given the setting of your research; please describe:

☐ Yes ☒ No **5. Will ISU students or other college students be asked to participate in your study?**

☐ Yes (see 5.a.(2)) ☐ No (see 5.a.(1)) **5.a.** If *Yes*, do you plan to ***include*** college students who may be under age 18?

5.a.(1) If *No (i.e., students under 18 will be* ***excluded*** *from your study)*, please describe how you will ensure college students under 18 do not participate in the study.

5.a.(2) If *Yes (i.e., students under 18 will be* ***included*** *in your study)*, please be sure to describe the parental consent and minor assent processes in Appendix E.

PART E: RECRUITMENT PROCEDURES

1. How will you identify or search for potential participants? (Check all that apply.)

- ☐ Review of public records (e.g., voter lists, utilities lists, phone directory, ISU directory, etc.)
- ☐ Review of private records (e.g., medical records, student records, other private records)
- ☐ Purchased mailing lists
- ☐ Personal contacts/knowledge
- ☐ "Snowball" sampling
- ☒ Participant responses to posted advertisements (electronic or hardcopy) or flyers
- ☒ Other; please describe: *We will contact individuals from a recruitment base of >400 adults who volunteered for our previous survey study and agreed to be contacted for future studies.*

2. Please describe the details of how each of the methods checked in #1 above will be implemented.
We will recruit exercise participants by posting advertisements around Iowa State buildings with permission, as well as send an email to ISU faculty and staff. Another email will be sent to those individuals from our database of previous study participants who agreed to be contacted for future studies and provided their eamil address. We believe these initial strategies of contacting school staff, along with word-of-mouth and $40 participation incentive per individual will be effective for recruiting. Please see attached for our recruitment flyer.

3. What methods will you use to contact potential participants? (Check all that apply.)

- [x] Letter or email
- [ ] Phone call
- [x] Posting flyers
- [ ] Posting announcement on website (Check all that apply.)
  - [ ] ISU Department of Psychology SONA system
  - [ ] ISU Department of Marketing/MIS SONA system
  - [ ] ISU Office of the Vice President for Research and Economic Development
  - [ ] ISU Departmental/Research Project websites
  - [ ] Other; please describe:
- [ ] Distribution of email or advertisement via Listserves or online bulletin-boards
- [ ] Television or radio advertisements
- [ ] Personal or verbal announcement, such as in a class, meeting, etc.
- [ ] Informal, personal communication
- [ ] Other; please describe:

4. Please describe the details of how each of the methods checked in #3 above will be implemented.
Emails will be sent to Iowa State University faculty and staff members, as well as individuals from our previous study who volunteered to be e-mailed about future studies. Advertisements will also be posted around Iowa State University after gaining permission.

☒ Yes	☐ No	5. Attached are copies of any letters, emails, phone/verbal scripts, flyers, announcements, or advertisements that will be used. Please know the IRB must review final and complete copies of all materials used to contact or recruit subjects. For verbal processes, a script or list of points to be covered during the discussion must be provided.
		If *No*, please explain why:

PART F: SCREENING PROCEDURES

☒ Yes	☐ No	1. Will participants be asked to provide any information about themselves (e.g., medical history, personal characteristics) for screening purposes prior to enrollment in the study?
		If *Yes*, please describe:<br><br>Yes, participants will complete a Medical History questionnaire (attached) and Physical

		Activity Readiness Questionnaire (PAR-Q. attached) prior to enrollment in the study. In addition, participants aged 70-74 years will be referred to their physician to check if they can participate in the study, as instructed by the PAR-Q. This will help to ensure that participants are not at high risk of adverse events.
☒ Yes ☐ No	**2.**	**Will participants be asked to take part in any interventions (e.g., fasting, blood draws, etc.) for screening purposes prior to enrollment in the study?**
		If *Yes*, please describe:<br><br>Participants will be seen on 4 occasions (1 orientation and 3 education sessions) prior to randomziation for the exercise protocols and the start of the exercise intervention. During these visits, participants are required to have an average systolic blood pressure between 120-159 mm Hg or average diastolic blood pressure between 80-99 mm Hg as this is the main outcome measure of the study. Participants will also complete a body composition analysis via bioelectrical impedence method, which has no reported bodily harm or injury, to determine if their body mass index lies in the range of 25-40 kg/m2.<br><br>However, there is no interventions such as fasting or blood draws for screeing purposes prior to enrollment in the study.

3. If *Yes* to question 1 and/or 2, please describe how you will obtain the informed consent of participants PRIOR to their participation in screening activities.

Participants will sign our informed consent document (attached) pertaining to the entire study prior to starting the medical history questionnaire during the orientation session, which is the first meeting with potential participants. If they would choose to not want to participate in the study, the participant would not complete any of the screening procedures.

PART G: COMPENSATION

☒ Yes ☐ No	**1.**	**Will participants receive any of the following types of compensation for their participation in your research? (Check all that apply.)**<br>☒ Money (cash or check)<br>☐ Gift cards<br>☐ Gifts<br>☐ Reimbursement for expenses (i.e., costs of travel to lab, child care, meals, etc.)<br>☐ Course credit (including extra credit)<br>☐ Other; specify:
	**2.**	If *Yes*, please answer questions 2a through 2d. ***This information should also be provided in the informed consent document.***
		**2.a.** Describe the specific amount of compensation to be provided (i.e., in monetary terms, points for course credit, value of gifts, etc.).

An honorarium in the form of cash of $40.

	**2.b.** Explain how compensation will be provided if the participant withdraws prior to completion of the study. **Note:** Completion of all study procedures cannot be a requirement for research participants to receive compensation.
	Each participant will receive $20 after completion of the baseline examination and another $20 after completing the 12-week post-intervention examination to promote both the adherence to the exercise program and to complete the follow-up examination. If an individual withdraws prior to the 12-week examination, they will not receive the second half of the compensation.
	**2.c.** If course credit is given, describe alternative ways students can earn the same amount of credit and how these alternatives are *genuinely comparable* to participation in the study in terms of time and effort.
	**2.d.** If the study involves multiple visits, sessions, or time-points, how will compensation be prorated (e.g., how much will be provided per visit/session/time-point)?
	The compensation will not be prorated by exercise session. We will be providing half of the compensation at each examination (baseline and 12-weeks).
	**Note: Compensation plans must be in accordance with policies set forth by the ISU Controller's Department. Detailed information is available here.**

PART H: RESEARCH PLAN

**1. Research Procedures – Using *layperson's terminology*, please describe in detail your plans for collecting data from participants. Include a description of all *procedures, tasks, or interventions* participants will be asked to complete during the research (e.g., random assignment, any conditions or treatment groups into which participants will be divided, mail survey or interview procedures, observation protocols, sensors to be worn, amount of blood drawn, etc.).**<br><br>**Note: When referencing attached documents (i.e., surveys, interview protocols, copies of stimuli, instructions for tasks, etc.), please ensure that each attachment is clearly labeled and clearly referenced in this section.**
In this 12-week randomized controlled trial on the effect of exercise on blood pressure, we will collect data from participants and there are 4 data collection points: 1) orientation session, 2) education sessions, 3) baseline examination and randomization, and 4) 12 week post-intervention examination.<br><br>Orientation session (approximately 60 minutes): After completion of recruitment, each participant will attend an orientation. During this orientation, we will explain the study and present the informed consent. Based on the questionnaires used by large epidemiological studies, we developed a questionnaire in layperson's terminology pertaining to the participants lifestyle, medical history, health status, and current physical activity. Participants will complete this questionnaire (attached). At this time, each participant will also complete the Physical Activity Readiness Questionnaire (attached) to assure there are no medical risks we should be aware of. Measurements will

also be taken of resting blood pressure and body composition in a private room to screen participants' eligibility.

Three education sessions (approximately 40 minutes in each session): After orientation, eligible participants will be asked to attend 3 education sessions over a week to provide lifestyle education and determine participants' ability to come to center 3 times per week, which will help minimize drop-out rate and increase compliance. During education sessions, we will measure resting blood pressure in each visit and the average blood pressure from orientation and education sessions will be used to screen eligibility.

Two baseline examinations and randomization (approximately 20 minutes in the first day examination and 60 minutes in the second day examination): The first day of the baseline examination will consist of the participant coming to Forker in the morning for a 12-hour fasted blood draw by a trained and experienced nurse. Approximately 5 ml of blood, which corresponds to about 1 teaspoon, will be drawn from a superficial arm vein and sent to a commercial lab for analysis (Labcorp, Des Moines, IA). We will measure a basic blood chemistry profile including plasma fasting glucose and lipids (total cholesterol, low- and high-density lipoprotein cholesterol, and triglyceride). The second day baseline measurement includs resting blood pressure and heart rate, body composition, and physical fitness tests (treadmill and strenght tests). Detailed descriptions for each measurement are explained below. At the end of the second day baseline examination, participants will receive physical activity log (attached), pedometers, and a 3-day diet log (attached) to collect their baseline physical activity and diet data. After one week, participants in both exericse and control groups will submit their physical activity log and 3-day diet log to the lab. However, all participants in both exercise and control groups will wear a pedometer throughout the study for 12 weeks and submit their pedometer diary (attached) and pedometer at the end of the study after 12 weeks.

* Resting blood pressure and resting heart rate will be measured after at least a 10-minute period of seated rest using an automatic digital blood pressure monitor with the upper left arm bared without constrictive clothing, legs uncrossed, and back and arm supported. A minimum of 2 blood pressure measurements will be taken at intervals of at least 2 minutes. If there is >5 mm Hg difference between the 1st and 2nd readings, an additional 1 or 2 readings will be obtained, and the average of 2 or more readings will be recorded following the American Heart Assoication recommendations.
* Body composition will be measured using bio-electrical impedence analysis. With the Tanita SC-331S, weight (kg), body mass index (kg/m2), body fat percentage (%), fat mass (kg), and fat free mass (kg) will be calculated. This occurs from the 8 electrodes beneath the participant's feet while standing on the device, in which they will not be wearing shoes or heavy clothing. We will also use skin fold measurements to analyze body fat, with all measurements taken on the right side of the body. Men will be measured at the chest, abdomen and thigh, whereas women will be measured at the tricep, thigh, and suprailliac. Each measurement will be assessed 3 times and averaged. Using gender-specific equations, we will be able to quantify the percentage body fat from these readings. Height (m) will be measured with a standard stadiometer, without shoes. Waist circumference (cm) will be taken at the umbilicus level and recorded with light clothes, and reading of the measurement will be taken at the end of exhaling.
* Cardiorespiratory fitness will be estimated using the submaximal treadmill test based on the Modified Balke and Ware Protocol used in the Aerobics Center Longitudinal Study. This protocol is safe and appropriate for non-athletic populations including older adults, deconditioned, and/or diseased individuals. Also, due to its smaller increments (e.g., 1% grade increase per minutes), it is a suitable protocol for estimating maximal grade and speed based on individual change in heart rate as exercise intensity increases. This protocol requires participants to initially begin walking on the treadmill at 3.3 miles per hour with a 0% incline (grade) for 1 minute, then 2% incline after 1 minute. After 2 minutes, the treadmill grade increases 1% every minute with the speed fixed at 3.3 mph until the participants reach 85% of their age-predicted maximal heart rate (220-age), or the certified exercise specialist will stop the test based on indications for terminating exercise testing following the American College of Sports Medicine (ACSM) guidelines including any adverse signs or symptoms, or the participants request to stop. We expect most participants would reach 85% of their age-predicted maximal heart rate before 20 minutes in this submaximal test of middle-aged population based on the average maximal treadmill time of 18 minutes in a large study of over 80,000 middle-aged adults from the Aerobics Center Longitudinal Study, in which the same treadmill protocol was utilized. Heart rate and perceived exertion (using the 6-20 category Borg's scale) will be monitored near the end of each minute. In addition, participant's appearance and symptoms will be monitored and recorded regularly. All participants will be familiarized with the treadmill and protocol before the baseline test, and a cool-down or recovery period for at least 5 minutes will be provided. Cardiorespiratory fitness in VO2max will be estimated using the following formula from the ACSM: 3.5 + (0.1 X speed) + (1.8 X speed X grade) from the estimated highest speed and grade that would have been achieved if the participant had worked to maximum.
*Muscular strength will be calculated for both upper and lower body by a one repetition maximum (1RM) protocol using the bench and leg press, respectively, with weight machines in the Physical Epidemiology Lab in the Forker building. Upon being familiarized with the equipment, taught proper technique, and an adequate warm-up is performed, each participant will do several submaximal repetitions with a spotter. Next, 50-70% of the individual's estimated 1 RM will be selected. Upon completion of 1 full repetition through the entire range of motion, weight will

progressively be increased by 5 pounds until the participant cannot successfully complete a full repetition. The final weight lifted successfully will be considered the participant's absolute 1RM.

Randomization: After baseline examinations, eligible participants will be randomly allocated in equal numbers (20 participants in each group) to 1) aerobic training only group, 2) resistance training only group, and 3) combination of both aerobic and resistance training group, and 4) no-training waiting list control group who will receive free exercise program after study. Thus, neither the researchers nor the participants can choose which group. Using a computer-automated system, randomization will be stratified by sex to promote an equal number of males and females to each group. Also, the same method will be used for age (45-54, 55-64, or 65-74 years). Thus, the distribution of sex and age will be similar across groups and the exercise training effect can be compared objectively. This randomization method will minimize an imbalance in covariates (sex and age) related to the outcome across groups and the exercise training effect will be least confounded by the covariates in this intervention trial.

Exercise programs: After randomization, each participant will start their assigned exercise protocol depending on their assignment:
1) Aerobic exercise only group: Train 3 days per week for 12 weeks. Each session will be 60 minutes in length in which they will ride the recumbant exercise bike or walk/jog on the treadmill at 50-80% of their VO2 Reserve. (=VO2 Max - resting VO2).
2) Resistance exercise only group: Train 3 days per week for 12 weeks. Each session will be 60 minutes in length in which they will perform 3 sets of 8-12 repetitions of 12 exercises for the major muscle groups (chest press, shoulder press, pull-down, lower-back extension, abdominal crunch, torso rotation, biceps curl, triceps extension, leg press, quadriceps extension, leg curl, and calf raise).
3) Aerobic and resistance exercise group: Train 3 days per week for 12 weeks. Each session will be 60 minutes, in which 30 minutes will be allocated to aerobic exercise, and 30 minutes allocated to resistance exercise. The protocol for the aerobic exercise will remain the same as above, but being half the time. As for resistance, they will perform 2 sets per exercise with 8-12 repetitions of only 9 exercises to account for the time difference.
4) Control group: No exercise training. These participants will refrain from any moderate-vigorous physical activity. They will submit copies of their activity logs and daily reported steps via the pedometer on a weekly basis.
Each participant will be taught proper technique for their assigned protocols prior to their first day of exercise by certified exercise professionals. Exercise professionals will also be observing participants at all times to ensure the safety of the participants and in attempt to minimize injury and other risks. These exercise professionals will also lead the 5 minute warm-up and cool-down before and after each session of exercise.

Upon completion of the 12-week protocol, each participant will go through a 12-week post-intervention examination over 2 days. This evaluation will follow the same protocol as the baseline evaluation and involve measurments of fasted blood draw, resting blood pressure and heart rate, body composition, cardiorespiratory fitness and strength tests. In addition, during the last 12th week of the study, all participants in both exerciser and control groups will complete their 7-day physical activity log and 3-day diet log and submit it to the lab during the 12-week post-intervention examination. All participants will receive 2 copies of both 7-day physical activity log and 3-day diet log at the baseline examination for both baseline and 12-week post-intervention physcial activity and diet data collection. At the end of the 11th week of the study, all participants will receive an eamil to remind of completing 7-day physical activity log and 3-day diet log. After entry of the 12-week evaluation data, all keys and personally identifiable information will be destroyed and removed from the data.

RESEARCH INVOLVING DECEPTION OR INCOMPLETE DISCLOSURE

☐ Yes ☒ No

2. Will participants be *deceived or misled* about anything during the study?
If *Yes*, please answer questions 2a through 2d in Appendix A.
If *No*, please skip to question 3.

☐ Yes ☒ No

3. Do you plan to *intentionally withhold* information from participants, such as the full purpose of the study, a full description of procedures, etc.?
If *Yes*, please answer questions 3a through 3d in Appendix A.
If *No*, please skip to question 4.

RESEARCH INVOLVING EXISTING DATA OR INFORMATION FROM RECORDS

☒ Yes ☐ No

4. Does the research involve the collection or study of currently existing data or information to be gathered from records, such as the following? (Check all that apply.)

☐ Student/educational records (including collection of class assignments, tests, etc.)
☐ Medical records (If checked, submit the Application for Use of Protected Health Information.)
☒ Data collected for a previously conducted study
☐ Information from government databases, such as the US Census, Iowa Dept. of Public Health records, etc.
☐ Samples from specimen/tissue banks
☐ Other; please describe:

If *Yes*, please answer questions 4a through 4g in Appendix B.
If *No*, please skip to question 5.

RESEARCH INVOLVING OBSERVATION

☐ Yes ☒ No

5. Does the research involve collection of data from observation of people's behaviors or activities?
If *Yes*, please answer 5a through 5d in Appendix C.
If *No*, please skip to question 6.

RESEARCH INVOLVING INTERNATIONAL RESEARCH

☐ Yes ☒ No

6. Will the research take place in an international setting?
If *Yes*, please answer 6a through 6c in Appendix D.
If *No*, please skip to question 7.

RESEARCH INVOLVING INVESTIGATIONAL DRUGS, DEVICES, DEXA/CT SCANS, X-RAYS, OR HUMAN CELLS OR TISSUES

☐ Yes ☒ No **7. Does this project involve an investigational new drug (IND)?** Number:

☐ Yes ☒ No **8. Does this project involve an investigational device exemption (IDE)?** Number:

☐ Yes ☒ No **9. Does this project involve DEXA/CT scans or X-rays?**

☒ Yes ☐ No **10. Does this project involve human blood components, body fluids, or tissues?**

☐ Yes ☒ No **11. Does this project involve human cell or tissue cultures (primary or immortalized)?**

If you answered *Yes* to either question 10 or 11 and the cells, body fluids, etc., have not been documented to be free of blood-borne pathogens, personnel handling these substances are required to take Blood-borne Pathogens Training annually.

Bloodborne Pathogens training is online via the EH&S website.

If you have any questions, contact EH&S at (515) 294-5359.

PART I: DATA ANALYSIS

1. Describe how the data will be analyzed (*e.g., statistical methodology, statistical evaluation, statistical measures used to evaluate results*).
We will conduct the primary outcome analyses using the intention-to-treat principle and include all participants as randomized. Analyses will take into account covariates including age, sex, race, body mass index, and baseline values of each outcome meausre. For all available data, we will use linear mixed-effects models for repeated measures over time with effects for time, study group, and time-by-group interaction. Within the mixed model, we will estimate 95% confidence intervals and P values for the 6 pre-specified inter-group contrasts: (1) aerobic training vs. control, (2) resistance training vs. control, (3) aerobic training vs. resistance training, (4) combined training vs. aerobic training, (5) combined training vs. resistance training, and (6) combined training vs. control for changes in outcome variables between baseline and 12 weeks. Additionally, the potential effects of missing data (or participants who do not meet the criteria of greater than or equal to 80% adherence to their exercise prescription) will be explored through various imputation models. All P values will be two-sided and P <0.05 will be deemed significant using SAS software.

PART J: CONSENT PROCESS

According to federal regulations, participants can only be included in research if they, or their legally authorized representative, provide legally-effective informed consent. In some cases, the IRB can waive this requirement.

I. Consent for Adult Participants

☒ Yes ☐ No	**A. Will you obtain the informed consent of all participants?**
If A is *Yes*, please answer the following questions:	
	**1. Describe the procedures you will use to provide information about the details of the study to participants.**
	Potential participants will be able to view the inclusion and exclusion criteria, procedures, and assessments via the flyers and e-mails received. Participants will also be informed about the study, the description of all procedures, benefits and potential risks, and their right to stop participating via the informed consent document, prior to starting any measurement, the baseline examination, and signing the informed consent document.
	**2. Who, in general, will obtain informed consent from participants (i.e., explain the study, collect signed forms, etc.)?** Please do not list actual names of study staff; rather, describe their role such as "the principal investigator," "research assistants," etc.

	The principal investigator will train research assistants about informing and obtaining informed consent from participants and the principal investigator and research assistant will collect informed consent.
	**2.a.** What training have they received or will they receive regarding how to appropriately obtain informed consent?
	The principal investigator and all key personnel, including research assistants, have received complete training on the protection of human research participants, biomedical responsible conduct of research, and informed consent.
	**3. Information conveyed to participants must be in a language understandable to them. Please describe the measures you are taking to ensure the informed consent process is understandable (e.g., translation into another language, using commonly understood terminology, assessing reading level of the consent form, etc.).**
	Our informed consent was designed and developed for lay readers using commonly understood terminology. Therefore, with the anticipated higher and commonly used language, we don't expect any difficulties with understanding the informed consent document. In addition, according the the US Census data from 2007-2011, over 97% of the individuals 25+ in Ames are a high school graduate or have obtained higher education. With this, we don't anticipate there being any diffuculty reading the consent form.
	**3.a.** If translation is required, please provide the name of the person(s) who conducted the translation(s) and his/her qualifications for doing so.
	Language translation should not be necessary in this study as a majority of the potential partcipants in Ames are expected to speak English.
	**4. When will informed consent be obtained in relation to beginning data collection?**
	The informed consent document will be obtained after each participant reads and understands all sections of the consent and all of their questions have been answered, upon which they will sign the consent if they choose to participate in the study. This will occur prior to any questionnaire completion or measurements before the study.
☒ Yes ☐ No	**5. Will all participants sign a consent form to document the consent process? Note:** Signatures must be handwritten by the participant; typing one's name on a form does not constitute a legally valid signature according to federal regulations. If ***No***, please explain why.
☐ Yes ☒ No	**6. Do any of the researchers or key personnel involved in the study have a supervisory, evaluative, or other position of "power" over participants?** If ***Yes***, please describe the measures you are taking to minimize any coercion or undue influence (real or perceived).
☐ Yes ☒ No	**7. Are any participants likely to be unable to provide consent for themselves, such as**

	**those who have severe cognitive impairments, dementia, are in life-threatening situations, cannot communicate, etc.?** If *Yes*, please describe plans to obtain consent from the participant's legally authorized representative.
	**7.a.** To the extent possible, given the condition of the participant, how will you ensure they agree to take part in the research?
If A is ***No***, (i.e., you will NOT obtain informed consent from all participants), please answer the following:	
**8.**	**Please provide strong and compelling justification for why you cannot carry out your study if you had to obtain informed consent. Note:** The fact that obtaining consent would be inconvenient or time consuming is **not** considered to be sufficient justification.
**9.**	**Please explain why participants' rights and welfare will not be adversely affected if you do not obtain their consent.**

II. Parent/Legal Guardian Consent and Child Assent (applies when participants are under age 18 or are considered to be children in the country where the research takes place)

☐ Yes ☒ No	**A. Does your study involve children?**
If A is *Yes*, please complete the questions in <u>Appendix E</u>.	

PART K: RISKS/DISCOMFORTS

☒ Yes ☐ No*<br>see 1.a.-1.g.	**1.** Are there any foreseeable risks or discomforts to participants from taking part in your research? *If *No*, please answer the following question.
	If *No (i.e., there are no foreseeable risks or discomforts to participants)*, please explain why you believe this is the case:
	If *Yes*, please answer *Yes* or *No* to items 1.a through 1.g below. Indicate whether the following types of risks/discomforts are foreseeable. When *Yes*, please describe the

risks/discomforts and explain how each will be mitigated or minimized.		
☒ Yes	☐ No	1.a. Physical Risks (e.g., injury, bruising from a blood draw, pain, side-effects from drugs administered, allergic reactions, etc.)
		Participants may experience discomfort or bruising from the blood drawing procedure during the basline and 12-week examination, in which we will attempt to minimize by having professional nurses complete the blood draws.<br>Though the risk of injury during exercise is low, injury to the muscles, ligaments, tendons, and joints of the body may occur due to the exercise involved with this research. We will attempt to minimize this issue by making sure all participants complete a thorough warm-up and cool-down prior to and after completing their exercise regimen. The participants will also only perform moderate-intensity exercise, reducing the risk compared to vigorous. In addition, there will be a gradual progression to minimize fatigue, soreness, injuries, and dropout.<br>Participants may experience abnormal blood pressure, fainting, dizziness, disorders of heart rhythm, and in very rare instances heart attack, stroke, or even death due to exercise participation. We will try to minimize any adverse outcomes by always monitoring participants during exercise, and allowing them to stop each exercise if they feel they cannot complete it. Participants will also complete a Physical Activity Readiness Questionnaire (PAR-Q) prior to exercise participation to make sure all are aware of the risks. In addition, any participants who are are high risk of adverse event will be refered to their physician before and throughout the study based on a comprehensive medical history questionnaire before the study, and signs/symptoms or report by participant during the exericse intervention.
☐ Yes	☒ No	1.b. Psychological Risks (e.g., emotional discomfort from answering questions, stress or anxiety from procedures, mood alterations, viewing offensive or "shocking" materials, etc.)
☐ Yes	☒ No	1.c. Social Risks (e.g., harm to reputation, embarrassment, or stigmatization if participation becomes known, disruption of personal or family relationships, etc.)
☐ Yes	☒ No	1.d. Economic Risks (e.g., loss of money, loss of or harm to employment, etc.)
☐ Yes	☒ No	1.e. Legal Risks (e.g., criminal liability if information about participants' illegal behaviors is collected)
☐ Yes	☒ No	1.f. Informational Risks (e.g., harm if information collected about the participant were disclosed or overheard, such as embarrassment, retribution, stigmatization, disruption of personal relationships, legal liability, etc.)
☐ Yes	☒ No	1.g. Other Risks, given the setting of your research

PART L: PRIVACY AND CONFIDENTIALITY

1. Describe how participants' privacy will be protected during recruitment and data collection (e.g., discussions/procedures will be conducted in private locations, messages regarding the research will not be left on answering machines without permission of participant, documents or recordings will be kept secure, etc.).

All baseline and 12-week examination data will be collected on a one-on-one basis at the ISU research laboratory. After the initial baseline questionnaire completion, data from the questionnaire will be entered into the computer database, and each partcipant will be assigned a number for identification purposes. In addition, all electronic data will be entered into a password protected computer and password protected file following Iowa State policies. All paper instruments will be kept for reference in locked filing cabinets.

2. Please answer the following questions to describe the methods you will employ to maintain confidentiality and security of the data at all points in the research process (e.g., during data collection, during analysis, etc.):

2.a. Who will have access to the data and study records?

The principal investigator and other key personnel listed in this IRB application will have access to the data.

2.b. Describe how/where physical copies (i.e., paper files, samples, etc.) of data and study records will be stored (e.g., in cabinets, desks, shelves, etc.).

All physical data in paper forms will be kept in locked filing cabinets in the principal investigator's office.

2.c. Describe security measures in place to maintain security of physical/paper data, samples, or study records (e.g., how access will be controlled, locks, etc.).

To maintain security of data and study records, the principal investigator will have complete control of filing cabinets locked in his office.

2.d. Describe how/where electronic data will be stored (e.g., a desktop computer, laptop, portable drive, shared drive, etc.).

All electronic data will be stored in the principal investigator's ISU registered computer in his office.

2.e. Describe the measures in place to maintain security of electronic data (e.g., encryption, password-protection, firewalls, using university controlled systems, etc.).

All electronic data will be password protected using systems controlled by ISU.

☐ Yes ☒ No **2.f. Will your data include any audio recordings and/or video recordings of participants?**

	If *Yes*, please answer the following:
	**2.f.(1)** Who will have access to the audio and/or video recordings?
	**2.f.(2)** Describe how/where the audio and/or video recordings will be stored (e.g., in a cabinet, on a computer, etc.).
	**2.f.(3)** Describe the measures in place to maintain security and confidentiality of the audio and/or video recordings (e.g., how access will be controlled, locks, password protection, firewalls, etc.).
☐ Yes ☐ No	**2.f.(4)** Will the actual recordings or images of participants from recordings be shared in any dissemination (e.g., manuscripts, reports, presentations, etc.) of the study results? If *Yes*, what measures will you take to disguise their identity (i.e., blurring facial images, voice alteration methods, etc.)?
☒ Yes ☐ No	**2.g.** Will any identifiers or identifiable information (e.g., names, social security numbers, addresses, phone numbers, exact dates of birth, etc.) be collected with or linked to the study data at **any point in time?** If *Yes*, please answer the following:
	**2.g.(1)** Describe the identifiers that will be collected or linked to the study data.
	We will collect name, home address, phone number, and email address. However, we will not collect social security numbers and dates of birth.
	**2.g.(2)** Why is it necessary to collect identifiers or link identifiers to the study data?
	These identifiers are necessary for correspondance pertaining to when the participant is available each week to perform their exercise, as well as if there is a change in schedules.
	**2.g.(3)** At what point in the process will identifiers be separated or removed from the data?
	Identifiers will be removed from the electronic data after completion of the 12-week examination. This removal will eliminate the connection between data and each participant's personally identifiable information. We will keep a master list of the identifiers separately not linked to the data or identification numbers for contact purposes for future studies.
	**2.g.(4)** Please describe any coding systems you will use to maintain confidentiality of identifiable data (e.g., plans to replace names with ID codes or pseudonyms).

Each name will be replaced with a number (1-80) to identify participants, as well as the initials of their first and last name.

☒ **Yes** ☐ **No** **2.g.(5)** Will you create a "key" linking identifiers with any ID codes or pseudonyms?

If ***Yes***, how will you maintain control of the key and ensure the key is kept secure? **Note:** Best practice is to store the key in a separate location from the study data.

We will keep the participant's key (ID number code) separately in the principal investigator's filing cabinet.

At what point will the key be destroyed?

The key will be destroyed upon completion of the 12-week examinations.

☐ **Yes** ☒ **No** **2.h.** Have you or will you obtain a Federal Certificate of Confidentiality for this study? If ***Yes***, please submit a copy of the certificate materials with this application. **Note:** Certificates of Confidentiality are designed to protect identifiable research records against forced disclosure (e.g., subpoena). Certificates can be sought from the National Institutes of Health in certain circumstances. Visit the **Certificates of Confidentiality Kiosk for more information.**

☐ **Yes** ☒ **No** **2.i.** Will the data be shared or submitted to a repository or registry, such as the Clinical Trial Registry Databank (ClinicalTrials.gov), the Database of Genotypes or Phenotypes, or via other data sharing agreements? If ***Yes***, please describe.

3. What specific steps will you take to ensure participants are not identifiable (directly or indirectly via "deductive disclosure") when research results are reported?

Each participant will only be identified via a number and initials when the data is stored and reported, upon which the key will be destroyed after completion of the 12 week examination. Personal identifiable information unlinked to the data will only be used for contact purposes for the 12-week study, in which it will be destroyed upon completion.

☒ **Yes** **4.** Please check here to confirm that you will retain research records (i.e., signed consent forms, approved IRB applications, etc.) for at least 3 years after the study is complete. Doing so is required by federal regulations.

PART M: REGISTRY PROJECTS

☒ **Yes** ☐ **No** **1. Does this project establish a registry or databank?**

Note: To be considered a registry or databank: (1) the individuals whose data are in the registry/databank might be contacted in the future; and/or (2) the names and/or data pertaining to the individuals in the registry/databank might be used by investigators other than the one maintaining the registry/databank.

If *Yes*, please answer the following questions:
**1.a. What information/data will be included in the registry?**
E-mail address only
**1.b. What is the reason for establishing a registry (i.e., how will data from the registry be used)?**
We plan to establish a databank in order to contact particpants in the future for participation in a similar studies.
**1.c. Who will be involved in establishing and providing oversight of the registry?**
The principal investigator will establish and keep the databank password protected on his ISU registered computer.
☐ **Yes** ☒ **No** **1.d. Will the data in the registry be available to anyone other than the investigator(s) who maintain the registry?**

B. RESEARCH INVOLVING EXISTING DATA OR INFORMATION FROM RECORDS

Continuation from Part H: #4:

4.a. What is/are the source(s) of the data/records?

The source is a previous database from a cross-sectional study on physical activity in approximately 400 adults that was collected the summer of 2013. (IRB ID: 13-001)

☐ Yes ☒ No — **4.b.** Are *all of* the data/records **publicly** available, without restriction?

4.c. Describe the specific variables, information, or content that will be obtained from the data/records.

E-mail address

☐ Yes ☒ No — **4.d.** Is the use of the data/records subject to any restrictions, such as the following? (Check all that apply.)

- ☐ FERPA—The Family Educational Rights and Privacy Act (applies to student records)
- ☐ HIPAA—The Health Insurance Portability and Accountability Act (applies to medical records) – *If checked, submit the Application for Use of Protected Health Information*.
- ☐ Institutional policies (for personnel records or other private records)
- ☐ Confidentiality provisions promised to the persons whose data you will obtain, such as those described in previously signed informed consent documents
- ☐ Other; please describe:

4.d.(1) If *Yes*, please describe how you will meet or address those restrictions when obtaining the data.

☒ Yes ☐ No — **4.e.** Will any of the following identifiers be included with the information you obtain from these records? (Check all that apply.)

- ☐ Names: ☐ First Name Only ☐ Last Name Only ☐ First and Last Name
- ☐ Phone/fax numbers
- ☐ ID codes that can be linked to the identity of the participant (e.g., student IDs, medical record numbers, account numbers, study-specific codes, etc.)
- ☒ Addresses (email or physical)
- ☐ Social security numbers
- ☐ Exact dates of birth
- ☐ IP addresses
- ☐ Photographs or video recordings
- ☐ Other; please specify:

☐ Yes ☒ No — **4.f.** Is there a reasonable possibility that participants' identities could be ascertained from any combination of information in the data? If *Yes*, please describe:

☐ Yes ☒ No	**4.g.** Will you obtain the permission/consent of the persons to whom the data/records pertain to use their information in your research?
	**4.g.(1)** If *Yes*, please describe this process.
	**4.g.(2)** If *No*, please provide **strong** justification for why obtaining permission/consent is not necessary or not possible. **Note:** The fact that obtaining consent would be inconvenient or time consuming is **not** considered to be sufficient justification.
	Consent has already been obtained from when they voluntarily included their e-mail address when the study was conducted previously to be contacted for future research and signed the required form.
☐ Attached	**4.g.(3)** If access to the data/records is subject to any restrictions, please attach documentation from the record holder indicating that you may have access to the data/records without the written consent of the participant.

Continue to Part H: #5 (Observation)

IOWA STATE UNIVERSITY
OF SCIENCE AND TECHNOLOGY

Institutional Review Board
Office for Responsible Research
Vice President for Research
1138 Pearson Hall
Ames, Iowa 50011-2207
515 294-4566
FAX 515 294-4267

Date: 8/11/2014

To: Dr. Duck-Chul Lee
251 Forker Bldg

CC: Dr. Lorraine Lanningham-Foster
220 MacKay Hall

From: Office for Responsible Research

Title: Independent and Combined Effects of Aerobic and Resistance Training on Blood Pressure

IRB ID: 14-330

Approval Date: 8/8/2014 **Date for Continuing Review:** 7/14/2016

Submission Type: Modification **Review Type:** Expedited

The project referenced above has received approval from the Institutional Review Board (IRB) at Iowa State University according to the dates shown above. Please refer to the IRB ID number shown above in all correspondence regarding this study.

To ensure compliance with federal regulations (45 CFR 46 & 21 CFR 56), please be sure to:

- **Use only the approved study materials** in your research, **including the recruitment materials and informed consent documents that have the IRB approval stamp.**
- **Retain signed informed consent documents for 3 years after the close of the study**, when documented consent is required.
- **Obtain IRB approval prior to implementing <u>any</u> changes** to the study by submitting a Modification Form for Non-Exempt Research or Amendment for Personnel Changes form, as necessary.
- **Immediately inform the IRB of** (1) **all serious and/or unexpected adverse experiences** involving risks to subjects or others; and (2) **any other unanticipated problems involving risks** to subjects or others.
- **Stop all research activity if IRB approval lapses**, unless continuation is necessary to prevent harm to research participants. Research activity can resume once IRB approval is reestablished.
- **Complete a new continuing review form** at least three to four weeks prior to the **date for continuing review** as noted above to provide sufficient time for the IRB to review and approve continuation of the study. We will send a courtesy reminder as this date approaches.

Please be aware that IRB approval means that you have met the requirements of federal regulations and ISU policies governing human subjects research. **Approval from other entities may also be needed.** For example, access to data from private records (e.g. student, medical, or employment records, etc.) that are protected by FERPA, HIPAA, or other confidentiality policies requires permission from the holders of those records. Similarly, for research conducted in institutions other than ISU (e.g., schools, other colleges or universities, medical facilities, companies, etc.), investigators must obtain permission from the institution(s) as required by their policies. **IRB approval in no way implies or guarantees that permission from these other entities will be granted.**

Upon completion of the project, please submit a Project Closure Form to the Office for Responsible Research, 1138 Pearson Hall, to officially close the project.

Please don't hesitate to contact us if you have questions or concerns at 515-294-4566 or IRB@iastate.edu.

Assigned IRB ID: **14-330**

INSTITUTIONAL REVIEW BOARD (IRB)
Modification Form for Non-Exempt Research

Title of Project: Independent and Combined Effects of Aerobic and Resistance Training on Blood Pressure			
Principal Investigator (PI): **Duck-chul Lee**			Degrees: Ph.D.
University ID: **937139203**	Phone: 515-294-8042	Email Address: dclee@iastate.edu	
Department: Kinesiology			

FOR STUDENT PROJECTS (*Required when the principal investigator is a student*)		
Name of Major Professor/Supervising Faculty:		
University ID:	Phone:	Email Address: @iastate.edu

Alternate Contact Person: Lorraine Lanningham-Foster	Email Address: lmlf@iastate.edu
Correspondence Address: 220 MacKay Hall	Phone: 515-294-4684

Please notify the IRB Office if your contact information has changed since the last review.

ASSURANCE

- I certify that the information provided in this application is complete and accurate and consistent with any proposal(s) submitted to external funding agencies. Misrepresentation of the research described in this or any other IRB application may constitute non-compliance with federal regulations and/or academic misconduct.
- I agree to provide proper surveillance of this project to ensure that the rights and welfare of the human subjects are protected. I will report any problems to the IRB. See Reporting Adverse Events and Unanticipated Problems for details.
- I agree that modifications to the approved project will not take place without prior review and approval by the IRB.
- I agree that the research will not take place without the receipt of permission from any cooperating institutions when applicable.
- I agree to obtain approval from other appropriate committees as needed for this project, such as the IACUC (if the research includes animals), the IBC (if the research involves biohazards), the Radiation Safety Committee (if the research involves x-rays or other radiation producing devices or procedures), etc., and to obtain background checks for staff when necessary.
- I understand that IRB approval of this project does not grant access to any facilities, materials, or data on which this research may depend. Such access must be granted by the unit with the relevant custodial authority.
- I agree that all activities will be performed in accordance with all applicable federal, state, local, and Iowa State University policies.

이덕철 8/31/2014

Signature of Principal Investigator   Date

Signature of Major Professor/Supervising Faculty   Date
(*Required when the principal investigator is a student*)

**For IRB Use Only**	Full Committee Review: ☐	Review Date: August 8, 2014
	Approval Not Required: ☐	Approval/Determination Date: August 8, 2014
EXEMPT per 45 CFR 46.101(b):	*Not Research*: ☐	Approval Expiration Date: July 14, 2016
EXPEDITED per 45 CFR 46.110(b): Category Letter 2	*No Human Subjects*: ☐	
	Not Approved: ☐	Risk: Minimal ☑ More than Minimal ☐
IRB Reviewer's Signature	Kerry A Agnitsch	August 8, 2014

Modification Information

The submission of a modification form is required whenever any changes are made to an approved project that requires expedited review or approval from the convened IRB. Modifications may include, but are not limited to,

- a change in the title;
- changes in investigators or key personnel;
- resubmission of a federal grant proposal involving changes to the original proposal;
- changes in the funding source (only when federal funding is involved);
- changes to data collection materials (e.g., informed consent documents, advertisements, survey or interview questions, etc.); or
- any other changes from the originally approved protocol (e.g., changes to confidentiality measures, inclusion/exclusion criteria, addition of an intervention or stimuli, etc.).

NOTE: All modifications must be approved by the IRB prior to implementation unless the change is necessary to protect the safety of participants.

Please provide answers to all questions, except as specified. The fields will expand as you type. Incomplete forms will be returned without review.

☐ Yes	☒ No	**Was your project initially determined to be eligible for exempt review?** *This information can be found in the approval letter you received when the study was last reviewed.*
		If *Yes*, **STOP!** This is not the correct form! Please submit a Modification Form for Exempt Research form instead.<br><br>If *No*, please complete Parts A and B below.

Part A: Changes in Personnel

☐ Yes	☒ No	**1.**	Does the modification involve a change in Principal Investigator? If *Yes*, **STOP!** The new principal investigator must submit a completed new Application for Approval of Research Involving Humans.
☐ Yes	☒ No	**2.**	Are you adding or removing members of the key personnel? If *Yes*, complete Table A.1 below.

Table A.1

1. **List any individuals who are no longer part of the key personnel:**

2. **Complete the following table to list any new key personnel:**

NAME	Interpersonal contact or communication with subjects, or access to private identifiable data?	Involved in the consent process?	Contact with human blood, specimens, or other biohazardous materials?	Other Roles in Research	Qualifications (i.e., special training, degrees, certifications, coursework, etc.)	Human Subjects Training Date
	☐	☐	☐			
	☐	☐	☐			
	☐	☐	☐			
	☐	☐	☐			
	☐	☐	☐			
	☐	☐	☐			
	☐	☐	☐			
	☐	☐	☐			
	☐	☐	☐			
	☐	☐	☐			
	☐	☐	☐			
	☐	☐	☐			
	☐	☐	☐			
	☐	☐	☐			
	☐	☐	☐			
	☐	☐	☐			
	☐	☐	☐			
	☐	☐	☐			
	☐	☐	☐			
	☐	☐	☐			

Please complete additional pages of key personnel as necessary.

☐ Yes ☐ No	**3. Do any of the individuals listed above have a conflict of interest management plan in place with the Office of the Vice President for Research & Economic Development?**
☐ Yes ☒ No	**4. Does your study include children (persons under age 18) as research subjects?**
	If *Yes*, please read and respond to the following:<br><br>ISU policy requires that background checks be completed for all researchers and key personnel who will have any contact with children involved in this research project. Details regarding this policy can be found here. **Principal Investigators and faculty supervisors are responsible** for ensuring that background checks are completed BEFORE researchers or key personnel may have any contact with children. Records documenting completion of the background checks must be kept with other research records (e.g., signed informed consent documents, approved IRB applications, etc.) and may be requested during any audits or Post-Approval Monitoring of your study.
☐ **Agreed**	**4.a. Please check here to indicate that you have read this information and agree that you will comply with these requirements.**

Part B: Administrative Modifications

☐ Yes ☒ No	1. Are there any changes to the project title?<br>If *Yes*, please specify new title:
☐Yes ☒ No ☐ N/A	2. Is there a change in funding source?
	If *No or N/A*, skip to Part C.<br><br>If *Yes*, please select from the following:<br>☐ Project is no longer funded. – *Proceed to Part C.*<br>☐ Project will have a new funding source. – *Please answer questions 2.a. through 2.g.*<br>☐ Project will have a change in funding source. – *Please answer questions 2.a. through 2.g.*
☐ Yes ☐ No	**2.a. Will this change result in the project having any external funding?**
	If *No*, skip to Part C.
	If *Yes*, please identify the type(s) of source(s) from which the project is directly funded.<br><br>☐ Federal agency<br>☐ State/local government agency<br>☐ University or school<br>☐ Foundation<br>☐ Other non-profit institution<br>☐ For-profit business

		☐ Other; specify:
☐ Yes	☐ No	**2.b. Is ISU considered to be the Lead or Prime awardee for this project?**
☐ Yes	☐ No	**2.c. Are there or will there be any subcontracts issued to others for this project?**
☐ Yes	☐ No	**2.d. Is or will this project be funded by a subcontract issued by another entity?**
☐ Yes	☐ No	**2.e. If ISU is the recipient of the subcontract, does it involve any federal funding, such as federal flow-through funds?**
**2.f. If this project will be externally funded, please provide the complete name(s) of the funding source(s); please do not use acronyms.** If any subcontracts will be issued to others, please describe and include a list of all entities.		
☐ **Attached**		**2.g. Please attach a <u>complete and final copy</u> of the entire new or revised grant proposal or contract from which the project is or will be funded.**

Part C: Protocol Modifications

**1. Please complete items 1.a. through 1.f. below to identify and describe all proposed modifications to your research procedures or study materials.**	
☒Yes ☐ No	1.a. Does the modification involve a change to the **research procedures**, such as the following? (Check all that apply.)
	☐ Method of data collection
	☐ Sources of data or records
	☐ Experimental design or conditions
	☐ Research interventions or stimuli
	☐ Recruitment methods of procedures
	☐ Inclusion/exclusion criteria or characteristics of participants
	☐ Number of participants
no change (ks)	☒ Compensation plans (including awarding course credit)
	☐ Confidentiality measures or privacy protections
	☒ Other; please specify: **Exercise intervention period change from 12 weeks to 8 weeks**
**1.b.** Please provide a detailed description of **each** change noted above in 1.a. The description should be complete, such that review of other documents (including attachments) is not required to understand the change.	
**Originally, the research was planned to have 12 weeks of exericse intervention, but we**	

now plan to change it to 8 weeks of the same exercise intervention without any changes in other research protocol including data collection, research design, exercise intervention program, inclusion/exclusion criteria, number of participants, compensation plan, or privacy protections.

* exercise frequency + duration will not increase - it will just occur for 8 weeks instead of 12 weeks. per 7/28/2014 email KH

☒ Attach a copy of any revised materials or documents with **all** changes clearly marked.

☒ Attach a final, "clean" copy of any revised materials or documents for inclusion in the file and the addition of an IRB approval stamp.

1.c. Explain the rationale for each proposed change:

After our original IRB application was approved, we had a meeting with other faculty members (Drs. Sharp and Franke) in our department to schedule the recruitment and exercise intervention, check all research places and facilities needed for data collection, and find additional research assistants, considering ongoing researches and classes in our department during this fall semester when the proposed research will be conducted. We also did a study simulation test and conducted a small pilot testing to check all places and times needed. Both faculty members suggested 8 weeks of exercise intervention instead of 12 weeks because of limited time and places to conduct the proposed study.

One specific limitation with 12 weeks of interventin is Thanksgiving break during the last stage of 12 weeks of intervention period that we can't control study participants' diet and lifestyle physical activity that may possibly influence our primary study outcome, which is blood pressure, and secondary outcomes such as blood lipids, glucose, body composition, and physical fitness. However, with 8 weeks of intervention, we will be able to complete exercise intervention before Thanksgiving break.

In addition, 8 weeks of exercise intervention was suggested as an appropriate exercsie intervention period that would have significant improvement on blood pressure (primary study outcome) in this pilot study to develop a large study proposal for external funding in the future.

We discussed this issue with all Key Personnel identified in the original IRB application including Dr. Lanningham-Foster who is a co-PI in the proposed study.

Drs. Sharp and Lanningham-Foster who approved the change of 8 weeks of intervention also previously reviewed the IRB application for this study as IRB committee members.

☒ Yes ☐ No **1.d.** Does the modification involve a change to the **study materials**, such as the following? (Check all that apply.)

- ☒ Recruitment materials
- ☒ Informed consent documents
- ☐ Survey instruments/questionnaires
- ☐ Interview or focus group questions or scripts
- ☐ Debriefing statements
- ☒ Other; please specify: **Recruitment Email and Doctor's permission form**

1.e. Please provide a detailed description of **each** change noted above in 1.d. The description should be complete, such that review of other documents (including attachments) is not required to understand the change.

**Only exercise intervention period was changed from 12 weeks to 8 weeks in all 4 study materials above.**
☒ Attach a copy of all revised materials or documents with all changes clearly marked.<br>☒ Attach a final, "clean" copy of all revised materials or documents for inclusion in the file and the addition of an IRB approval stamp.
**1.f.** Explain the rationale for each proposed change:
**To provide correct exercise intervention period to study participants in each document.**

If you have any questions or feedback, please contact the IRB office at IRB@iastate.edu or 515-294-4566.
